# Bacterial Single‐Cell Proteins as Sustainable Aquafeeds: A Meta‐Analysis of Growth, Physiological Homeostasis, and Antioxidant Capacity

**DOI:** 10.1155/anu/4548847

**Published:** 2026-04-25

**Authors:** Muziri Mugwanya, Fahad Kimera, Hani Sewilam

**Affiliations:** ^1^ Center for Applied Research on the Environment and Sustainability (CARES), The American University in Cairo, New Cairo, Cairo, Egypt, aucegypt.edu; ^2^ UNESCO Chair in Hydrological Changes and Water Resources Management, RWTH Aachen University, Aachen, North Rhine-Westphalia, Germany, rwth-aachen.de

**Keywords:** alternative proteins, aquafeed, feed utilization, microbial proteins, sustainability

## Abstract

In aquaculture nutrition, alternative protein sources, such as those derived from bacteria, have recently garnered significant attention as safe and viable replacements for traditional protein sources, including fish meal (FM) and soybean meal (SBM). This is attributed to their rich protein content and a well‐balanced amino acid profile that is comparable to that of FM. Likewise, their ease to be grown on industrial by‐products or gas fermentation warrants a continuous supply. Given their global importance in aquaculture nutrition and health, this study was conducted to quantify the overall effect of dietary inclusion of bacterial single‐cell proteins (BSCPs) on the specific growth rate (SGR), feed efficiency ratio (feed conversion ratio [FCR]), survival rate (SR), hepatosomatic index (HSI), viscerosomatic index (VSI), and biochemical responses (alanine aminotransferase [ALT], aspartate aminotransferase [AST], superoxide dismutase [SOD], and catalase [CAT] enzymatic activities) in several aquaculture species. Hedge’s *g* was computed to quantify the primary outcomes. Furthermore, the influence of several moderators, such as BSCP source, aquaculture species, and habitat, on Hedge’s *g* effect sizes was determined by a mixed‐effects model. Except for the SR, the results indicated nonstatistically significant differences in SGR, FCR, HSI, VSI, ALT, AST, SOD, and CAT between the BSCP treatment groups and the control, and this was uniform among carnivorous, omnivorous, and herbivorous species. The results of the mixed‐effects model revealed that the BSCP source and aquaculture species influenced the observed effect sizes of SOD and CAT. Similarly, BSCP source and habitat influenced the observed effect sizes for VSI. Overall, depending on the inclusion level, BSCPs are a safe and viable alternative to FM or SBMs, and their inclusion in aquaculture diets offers significant benefits.

## 1. Introduction

The aquaculture industry has recently undergone a tremendous transformation as a result of improved and diversified production techniques [1, 2]. However, this has led to an increase in demand for aquaculture feeds and ingredients, and reliable forecasts predict that any limitations in the supply chain will stall the industry’s growth [3]. Fish meal (FM), a nutrient‐rich protein source, has been the preferred ingredient; however, there have been debates regarding its environmental and economic concerns, warranting the need to partially or totally replace it in aquaculture diets [4–6]. As such, alternative protein sources have been proposed, among which are plant‐based proteins (i.e., soybean meal [SBM], cottonseed meal, sunflower meal, and rapeseed meal). However, the latter are not fully suitable for certain aquaculture species due to the presence of antinutritional factors, deficiencies in certain amino acids, high fiber and carbohydrates, and unpalatability [7–9]. Nonetheless, the replacement of FM with plant‐based proteins has been widely investigated, with inclusion levels ranging from 10% to 80%, exhibiting different degrees of efficacy in achieving an equilibrium between nutritional adequacy, safety, and sustainability [3, 10].

Most recently, however, the use of other protein alternatives, such as single‐cell proteins (i.e., microbial proteins extracted from pure or mixed cultures) derived from dried biomass of bacteria, yeasts, and microalgae in aquaculture nutrition is growing considerably due to their rich‐nutrient content (i.e., proteins, amino acids, and omega‐3‐fatty acids) and bioactive compounds like peptidoglycans and astaxanthin [11–13]. Of all single‐cell protein sources, there is a preference for bacteria due to their superior protein and amino acid content, and they have hence attracted more attention in the scientific community [14]. Moreover, their short generation time and efficiency in converting different substrates, such as hydrocarbons and carbon sources, into rich protein biomass make them feasible alternatives for industrial production [15]. Table 1 summarizes different substrates utilized by different bacterial species used in the industrial production of bacterial single‐cell protein (BSCP). The different methods of industrial production and bio‐economics of BSCPs have been extensively studied and presented in a review by Sharif et al. [27] and Gamboa‐Delgado and Marquez‐Reyes [28], respectively.

**Table 1:** Substrates used for culturing several bacterial species.

Bacterial species	Substrate	Reference
Purple non‐sulfur bacteria (PNSB)	Food waste	[16]
Purple phototrophic bacteria	Domestic wastewater	[17]
*Lactobacillus acidophilus*	Stickwater and glucose	[18]
*Clostridium autoethanogenum*	Industrial waste biomass	[19]
*Methylophilus methylotrophus*	Methane	[15]
*Methylomonas* sp.	Methane	[20]
Methane oxidizing bacteria	Methane	[21]
*Methylocapsa acidiphila*	Methane	[22]
*Methylobacterium organophilum*	Methanol	[15]
*Methanotroph capsulatus*	Methane	[23]
*Rhodopseudomonas* sp.	Digested sludge, small organic acids, and industrial wastewater components	[24, 25]
*Corynobacterium ammoniagenes*	Fructose, glucose	[26]

Recent research indicates that BSCP offers significant potential as an animal feed ingredient due to its high protein content (50%–75% dry weight basis) and digestibility and has been shown to enhance the growth performance and health status of aquaculture species [29–34]. Moreover, partial replacement of FM or SBM with BSCP has been shown to boost the growth and survival rate (SR) of several aquaculture species [35, 36]. For example, in an investigation by Chama et al. [37], FM was successfully replaced with BSCP derived from *Methylococcus capsulatus* (bath) in the feed for GIFT tilapia. The authors found no significant difference in key growth metrics such as the specific growth rate (SGR), feed conversion ratio (FCR), and SR between the BSCP treatment diets and the control diet. In another study, Liao et al. [38] showed that replacing FM with 4% or 8% BSCP (*Rhodobacter sphaeroides*) enhanced the hyposaline stress resistance of *Litopenaeus vannamei*. This dietary intervention led to improved growth (SGR and SR) and was associated with a favorable shift in gene expression, specifically upregulating immune and stress‐related genes (heat shock protein 70, superoxide dismutase [SOD], and relish), while downregulating the apoptosis gene, Caspase‐3. Likewise, Hardy et al. [35] reported improved SR in *Oncorhynchus mykiss* Walbaum upon replacement of SBM with 10% BSCP (*Methylobacterium extorquens*). These consistent findings at the cellular and organismal levels suggest a robust biological trend; however, the literature has now progressed beyond the stage where individual case studies suffice.

Whereas several high‐quality narrative reviews have expertly documented the qualitative landscape and production mechanisms of BSCPs [3, 19, 27, 28, 39], the field has reached a critical data density that warrants a transition from qualitative to quantitative analysis. To address this gap, a meta‐analysis was undertaken to systematically determine how dietary BSCPs influence growth performance (SGR and SR), feed utilization (FCR), somatic indices (hepatosomatic index [HSI] and viscerosomatic index [VSI]), and specific enzymatic activities (alanine aminotransferase [ALT], aspartate aminotransferase [AST], SOD, and catalase [CAT]). This study represents the first comprehensive meta‐analysis of BSCPs as sustainable alternatives to FM and SBMs. By conducting the first large‐scale meta‐analysis in this field, we transition from descriptive synthesis to a precise quantification of BSCP efficacy. This approach allows us to define the statistical boundaries of protein substitution and identify the key moderators that influence growth and physiological homeostasis.

## 2. Materials and Methods

### 2.1. Literature Search Strategy

We performed a systematic quantitative synthesis to evaluate the dietary inclusion of BSCPs across diverse aquatic taxa, focusing on an integrated suite of performance and health indicators. While growth (SGR and SR) and feed utilization (FCR) were prioritized for their commercial relevance, we further assessed somatic indices (HSI and VSI) and hepatic biomarkers (ALT and AST) to evaluate the metabolic costs and structural integrity of the liver. This multifaceted approach ensures that rapid growth does not compromise organ‐scale health or lead to abnormal lipid deposition. Additionally, the inclusion of antioxidant enzymes (SOD and CAT) provides a critical assessment of the primary physiological stress response and the maintenance of cellular homeostasis under novel dietary regimes.

**Figure 1:**
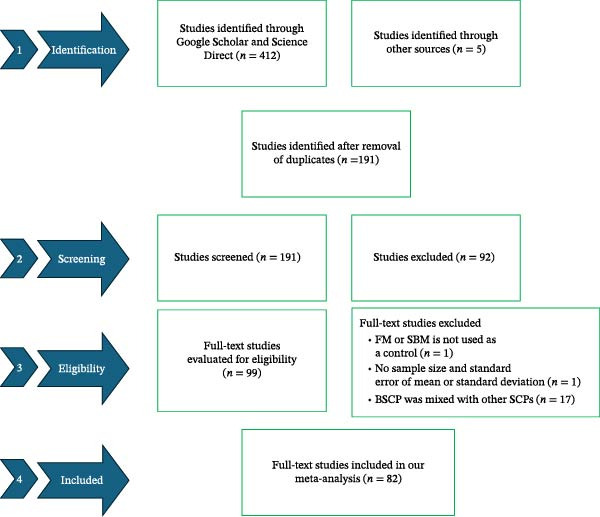
Flowchart of assessed and chosen studies following the PRISMA guidelines. BSCP, bacterial single‐cell protein; FM, fish meal; SBM, soybean meal; SCP, single‐cell proteins.

Our search, which adhered to the Preferred Reporting Items for Systematic Reviews and Meta‐analysis (PRISMA) guidelines, targeted the ScienceDirect and Google Scholar databases between September 3rd and 5th, 2025. The following search terms and keywords were applied to conduct the literature search: bacterial single‐cell protein OR methanogen bacterial single‐cell protein OR *Clostridium autoethanogenum* protein AND aquaculture AND growth performance. The internet search was exclusively limited to articles published in English and in peer‐reviewed journals. However, review articles, conference reports, dissertations, and theses were excluded from the study. To ensure comprehensive coverage, we also performed manual searches in addition to the database queries. The literature screening process was managed using Mendeley (desktop 1.19.8). Overall, ScienceDirect and Google Scholar generated 223 (*n* = 223) and 189 (*n* = 189) studies, respectively. Furthermore, our manual search generated five studies (*n* = 5). Figure 1 shows a flow chart of assessed and chosen studies following the PRISMA guidelines.

### 2.2. Inclusion and Exclusion Criteria

Our inclusion criteria was based on the following: (1) treatments were randomized in the feed trial; (2) studies that focused on fish and crustaceans; (3) studies where FM or SBM was replaced with BSCP with or without changes in key ingredients; (4) any of these indices; SGR, FCR, SR, HSI, VSI, ALT, AST, SOD, and CAT was measured in both control and experimental treatment groups; (5) average response, number of replicates, sample size, and an estimate of error (i.e. standard deviation [SD], standard error [SE], pooled SE [PSE]) was numerically reported or presented in graphs. Conversely, studies were excluded based on the following criteria: (1) no replication of treatments; (2) no given information on the sample size, SD, SE, and/or PSE; (3) SD of FCR exceeding 1.50; (4) no control diet; (5) FM or SBM not used as the control diet; (6) No dietary inclusion of BSCP; (7) none of these indices; SGR, FCR, SR, HSI, VSI, ALT, AST, SOD, and CAT was reported.

### 2.3. Data Collection

Out of 97 studies (*n* = 97) assessed for eligibility, 95 studies (*n* = 95) were selected for meta‐analysis based on our inclusion and exclusion criteria. Following an independent assessment of relevant data by two researchers, an Excel sheet was created to catalog the findings. The compiled information includes author names and year of publication, the BSCP source, characteristics of the aquaculture species (including habitat and feeding behavior), study design parameters (sample size and number of replicates per treatment), and key outcome measures (SGR, FCR, SR, HSI, VSI, AST, ALT, SOD, and CAT) with their associated estimates of error.

### 2.4. Data Analysis

Average values and SD for SGR, FCR, SR, HIS, VSI, ALT, AST, SOD, and CAT of the control and experimental treatment groups were extracted from the studies. In studies where the SE or PSE was used, the SD was calculated as follows:

SD= SE ×√n or SD= PSE×√n,

where *n* is the number of experimental replicates. Results that were presented in graphical form, mean, and SD values were obtained using an online tool, PlotDigitizer (https://plotdigitizer.com/).

The standardized mean difference (SMD) was calculated using Hedge’s *g* statistic because of its robust statistical power, its correction for small sample sizes in different experimental groups, and its ease of interpretation [40, 41]. To quantify the effect size, Hedge’s *g* was computed using common and random‐effects models for all measured parameters (SGR, FCR, SR, HSI, VSI, ALT, AST, SOD, and CAT). A positive *g* indicated a better outcome for the BSCP group compared to the control, while a negative *g* indicated a reverse outcome. Any *g* estimate that includes zero was considered to show no statistical difference between the BSCP group and the control.

Between‐study heterogeneity was assessed by computing the heterogeneity variance (*τ* ^2^) and the confidence interval (CI). Additionally, a sensitivity analysis incorporating the *I* ^2^ statistic [42] and the chi‐squared statistic (*Q*) was performed to further detect and quantify this variability [43–45]. To test the robustness of our meta‐analysis, a simple subset meta‐analysis was performed as described by Mathur [46], and the results were compared.

The influence of moderators, such as BSCP source, aquaculture species, and habitat, on the observed effect sizes for Hedge’s *g* was determined using a mixed‐effects model. Egger’s regression [47] and Begg’s rank correlation test [48] were used to detect publication bias. Egger’s regression test was adopted as a reference in case of inconsistent results between the two methods. When publication bias was detected, the Duval and Tweedie [49] trim and fill method was used to identify studies that influenced the outcome. Furthermore, Rosenberg’s fail‐safe number was calculated to test for publication bias in the datasets. All the statistical analyses were performed in R (version 4.4.1) using the metafor, esc, and meta packages.

## 3. Results and Discussion

### 3.1. Description of Studies Included in the Meta‐Analysis

After screening, a total of 95 (*n* = 95) studies were included in our meta‐analysis (Figure 1). From all these studies, 29 aquaculture species were reported, which included 17 carnivorous, 10 omnivorous, and two herbivorous species. The most studied carnivorous, omnivorous, and herbivorous aquaculture species were *Micropterus salmoides* (41.38%), *L. vannamei* (65.52%), and *Ctenopharyngodon idellus* (13.79%), respectively. Likewise, the most studied BSCP sources were *Clostridium autoethanogenum* (CAP) and *Methylococcus capsulatus*, Bath. These two bacterial species are widely used as alternative protein sources in aquaculture diets due to their high protein content (80% to 89% for CAP and 75.14% dry weight for *M. capsulatus*). Moreover, CAP has been reported to contain a lipid content of 0.2%–2.6% dry weight, with an amino acid composition similar to that of FM, and this is dependent on certain factors such as the culture substrate and fermentation process [19, 50].

### 3.2. SGR

**Figure 2:**
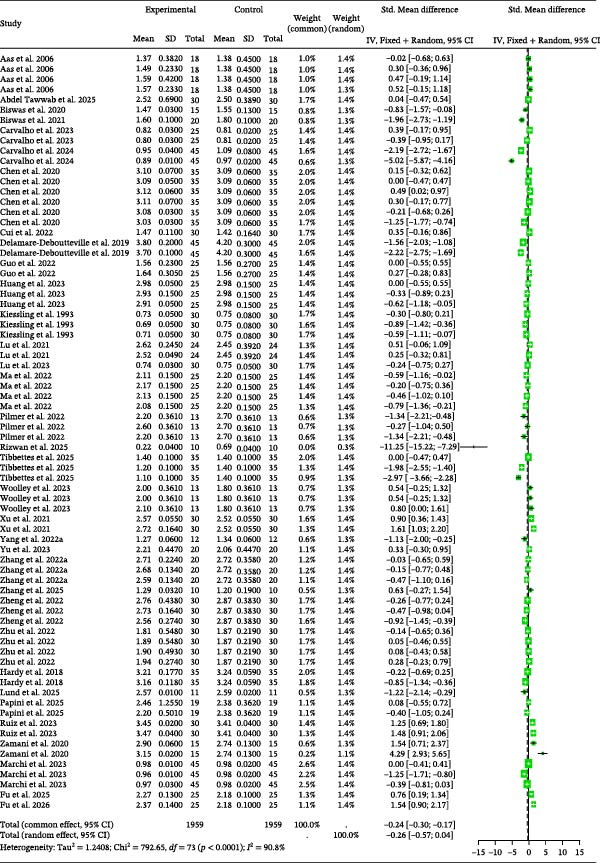
SGR forest plot of effect sizes of bacterial single‐cell proteins included in diets of carnivorous aquaculture species. Each square and size corresponds to the size of the mean effect and the relative weight of the related study, respectively. The vertical line represents the line of no effect, indicating no significant difference in the standardized mean difference.

Detailed parameters of 74 independent studies for the specific growth (SGR) are presented in Table S1. The meta‐analysis of SGR in carnivorous species (Figure 2) suggested that SGR was nonsignificantly higher in the control group than in the BSCP treatment group (Random Effects Hedge’s *g* = −0.27, *p* = 0.0913). The analysis detected high between‐study heterogeneity (*I* ^2^ = 90.8%, *p* < 0.0001), but found no evidence of publication bias (Table 2) or significant influence from tested moderators (BSCP source, species, and habitat; Table 3).

**Table 2:** Results of Egger’s regression test, Begg’s rank correlation test, and the trim and fill method for the detection of publication bias.

Growth and biochemical parameters	Egger’s regression test			Begg’s rank correlation test		Trim and fill method			
	Intercept	*t*‐Value	*p*‐Value	*z*‐Value	*p* ‐Value	Random effects model	*I* ^2^	*Q*‐ value	*p*‐Value
SGR (carnivorous species)	−1.0616	−0.59	0.5554	−0.00	0.9963	NA	NA	NA	NA
SGR (omnivorous and herbivorous species)	0.5132	0.21	0.8303	1.11	0.2691	NA	NA	NA	NA
FCR (carnivorous species)	0.3360	0.57	0.5731	1.17	0.2428	NA	NA	NA	NA
FCR (omnivorous and herbivorous species)	6.8179	3.84	0.0002	0.51	0.6098	−0.2566	97.3%	3438.00	0
Survival (carnivorous species)	0.9848	0.85	0.3986	1.14	0.2523	NA	NA	NA	NA
Survival (omnivorous and herbivorous species)	0.0118	0.01	0.9946	0.59	0.5582	NA	NA	NA	NA
HSI	−1.3782	−1.28	0.2052	−1.75	0.0794	NA	NA	NA	NA
VSI	−2.0612	−2.15	0.0358	−1.44	0.1495	0.1144	87%	600.68	<0.0001
AST	3.5937	1.52	0.1355	1.50	0.1337	NA	NA	NA	NA
ALT	−7.4749	−3.16	0.0027	2.98	0.0029	0.0513	96.4%	1652.51	<0.0001
SOD	6.5001	1.82	0.0760	−0.33	0.7451	NA	NA	NA	NA
CAT	14.9787	5.28	<0.0001	2.29	0.0221	0.2904	99%	2736.85	0

Abbreviations: ALT, alanine aminotransferase; AST, aspartate aminotransferase; CAT, catalase; FCR, feed conversion ratio; HSI, hepatosomatic index; NA, not applicable; SGR, specific growth rate; SOD, superoxide dismutase; SUR, survival; VSI, viscerosomatic index.

**Table 3:** Results for meta‐regression analysis of independent variables that influence the observed effect sizes of bacterial single‐cell proteins in aquaculture diets.

Growth and biochemical parameters	BSCP source				Aquaculture species				Habitat			
	*F*‐Value	*p*‐Value	*R* ^2^ (%)	Intercept	*F*‐Value	*p*‐Value	*R* ^2^ (%)	Intercept	*F*‐Value	*p*‐Value	*R* ^2^ (%)	Intercept
SGR (carnivorous species)	0.2969	0.5878	0.00	−0.0248	0.0000	0.9980	0.00	−0.2350	0.0111	0.9165	0.00	−0.1609
SGR (omnivorous and herbivorous species)	0.0482	0.8269	0.00	0.2903	0.0744	0.7858	0.00	0.3357	0.0541	0.8167	0.00	0.0086
FCR (carnivorous species)	2.6064	0.1133	3.75	1.1321	0.1418	0.7082	0.00	0.2783	0.7269	0.3983	0.00	1.1436
FCR (omnivorous and herbivorous species)	2.4124	0.1235	1.84	−0.9557	2.0486	0.1554	0.91	1.2473	2.6061	0.1096	1.38	−1.8278
Survival (carnivorous species)	3.9323	0.0569	11.54	−0.4901	0.7981	0.3790	0.00	0.3309	3.0506	0.0913	8.02	−1.0375
Survival (omnivorous and herbivorous species)	0.0530	0.8185	0.00	0.1136	0.8487	0.3600	0.00	0.2937	1.0339	0.3127	0.08	−0.1664
HSI	0.5592	0.4568	0.00	−0.1599	0.5623	0.4555	0.00	0.0920	3.2428	0.0755	2.97	−0.5420
VSI	13.6633	0.0005	21.49	−0.6543	0.2750	0.6019	0.00	−0.0668	0.0498	0.8242	0.00	−0.2244
AST	0.0352	0.8520	0.00	0.1933	0.0043	0.9480	0.00	0.1084	0.4942	0.4855	0.00	0.5110
ALT	0.2284	0.6350	0.00	−1.2954	2.7799	0.1021	6.68	−2.0372	0.0229	0.8803	0.00	−1.0021
SOD	14.5010	0.0005	26.00	−2.7549	5.4508	0.0247	10.24	1.7518	2.1770	0.1479	2.91	1.9119
CAT	19.7381	0.0002	45.32	−84.8535	2.8265	0.1063	7.24	31.2719	1.0425	0.3179	0.07	36.9765

Abbreviations: ALT, alanine aminotransferase; AST, aspartate aminotransferase; BSCP, bacterial single‐cell protein; CAT, catalase; FCR, feed conversion ratio; HSI, hepatosomatic index; NA, not applicable; SGR, specific growth rate; SOD, superoxide dismutase; SUR, survival; VSI, viscerosomatic index.

**Figure 3:**
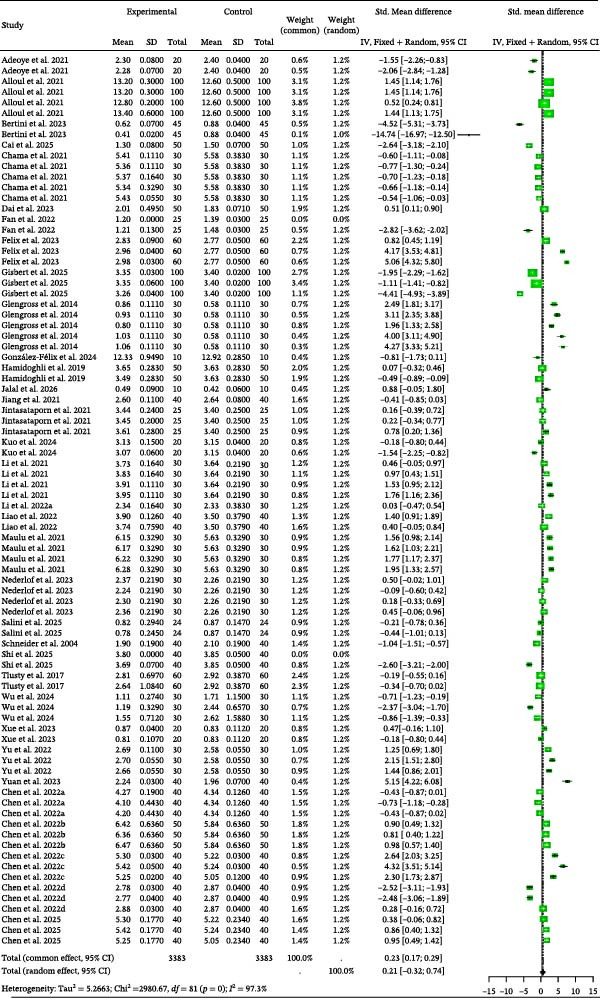
SGR forest plot of effect sizes of bacterial single‐cell proteins included in diets of omnivorous and herbivorous aquaculture species. Each square and size corresponds to the size of the mean effect and the relative weight of the related study, respectively. The vertical line represents the line of no effect, indicating no significant difference in the standardized mean difference.

Detailed parameters of 84 independent studies for the SGR of omnivorous and carnivorous species are presented in Table S2. The meta‐analysis of SGR (Figure 3) suggested that BSCP inclusion led to nonsignificantly higher growth than the control (random effects Hedge’s *g* = 0.21, *p* = 0.4347). This result was generated despite high between‐study heterogeneity (*I* ^2^ = 97.3%, *p* = 0). Furthermore, the data showed no indication of publication bias (Table 2) or significant explanatory influence from the tested moderators (BSCP source, species, and habitat; Table 3).

SGR is highly dependent on the dietary protein requirements of the reared aquaculture species [51, 52]. As the most vital macronutrient, protein supplies the amino acids (essential and nonessential) that fish require to build muscle and grow [53, 54]. The optimum protein level for aquaculture species is not fixed, but varies significantly depending on several factors, such as species, age, life stage, and rearing conditions. Studies indicate that SGR is maximized at this ideal protein level and diminished by either insufficient or excessive amounts [55–58]. It is imperative to note that carnivorous species have higher protein requirements that range from 40% to 55% [59–61] compared to those of omnivorous (25%–45%) [62–64] and herbivorous (25%–35%) [65, 66] species. Nonetheless, inclusion of BSCP in aquaculture diets provides the required proteins and amino acids needed for proper growth and tissue maintenance of aquaculture species. For instance, Carvalho et al. [67] reported an improved SGR in juvenile gilthead sea bream (*Sparus aurata*) after replacing 66% of FM with BSCP. In another study, Xue et al. [68] showed that substituting 30% of the SBM with BSCP (CAP) improved both the SGR and crude protein content in the muscles of grass carp (*Ctenopharyngodon Idella*). Similarly, Li et al. [69] documented a substantial increase in the SGR of Jian carp (*Cyprinus carpio* var. Jian) following the incorporation of BSCPs into their diets. BSCP proteins typically possess a crude protein content of 50%–75% by dry weight, matching or exceeding that of conventional FM [15, 50, 70]. Consequently, BSCPs offer an essential amino acid profile that closely aligns with the nutritional requirements of carnivorous fish. BSCPs are especially crucial for their rich content of lysine, methionine, and cysteine, amino acids frequently identified as limiting factors in plant‐based proteins [27]. In aquaculture, dietary lysine requirements typically vary from 1.3% to 2.2% of the dry diet, depending on the species and developmental stage [71], while methionine requirements range from 0.49% to 2.5% [72]. Furthermore, research indicates that cysteine can effectively substitute for a portion of the methionine requirement, with sparing effects generally observed between 40% and 60% [73, 74]. The positive correlation between the availability of these specific amino acids and the enhancement of SGR, as demonstrated in various studies [75, 76], provides a strong biochemical rationale for the improved growth performance observed in aquaculture species fed BSCP–supplemented diets.

### 3.3. FCR

**Figure 4:**
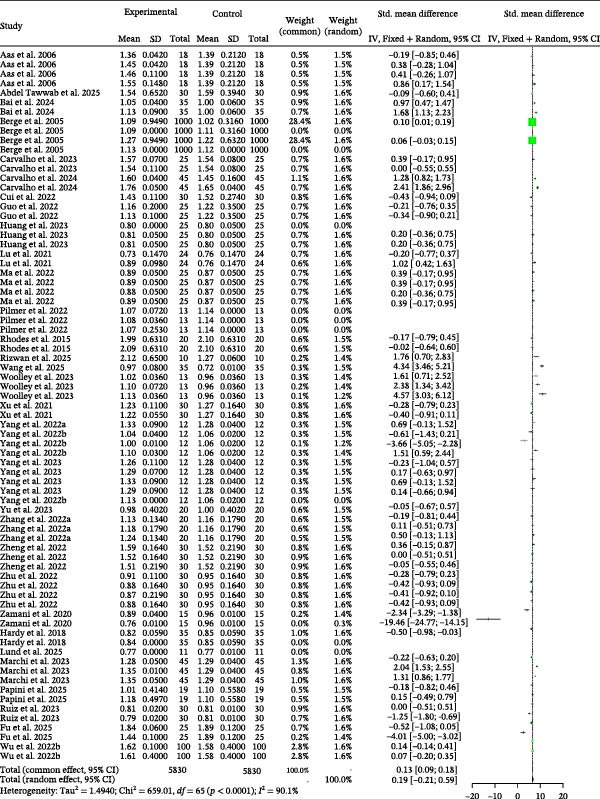
FCR forest plot of effect sizes of bacterial single‐cell proteins included in diets of carnivorous aquaculture species. Each square and size corresponds to the size of the mean effect and the relative weight of the related study, respectively. The vertical line represents the line of no effect, indicating no significant difference in the standardized mean difference.

Detailed parameters of 75 independent studies for the FCR of carnivorous species are presented in Table S3. The meta‐analysis found a trend toward higher FCR in the BSCP diets (poorer feed efficiency) compared to the control (Figure 4). The positive Hedge’s *g* values from both models confirmed this trend, though the effect was statistically nonsignificant in the random‐effects model (*g* = 0.19, *p* = 0.3557). The analysis highlighted significant heterogeneity across studies (*I* ^2^ = 90.1%, *p* < 0.0001), but found no evidence of publication bias (Table 2) or significant explanatory effect from the tested moderators (Table 3).

**Figure 5:**
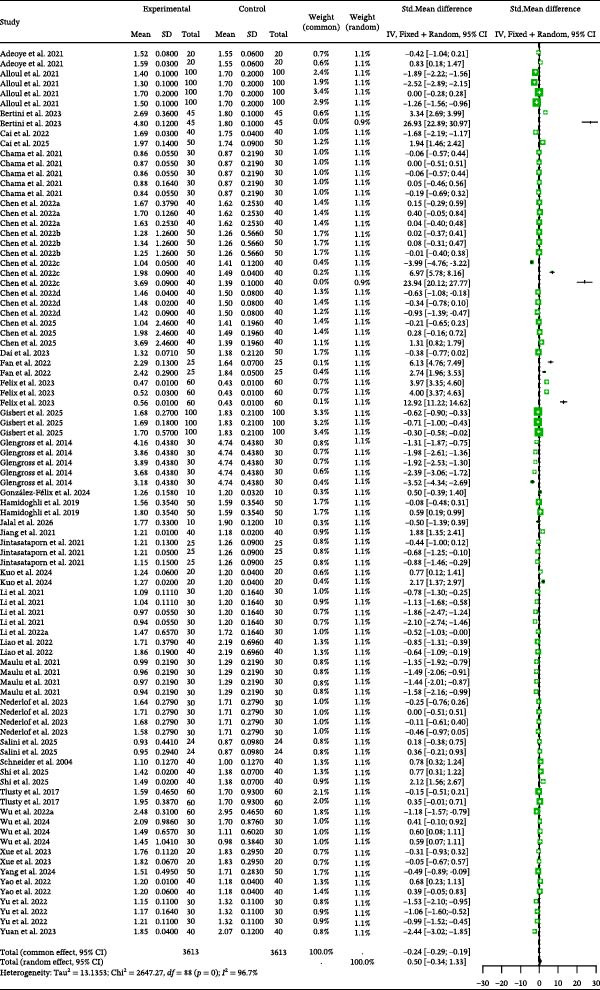
FCR forest plot of effect sizes of bacterial single‐cell proteins included in diets of omnivorous and herbivorous aquaculture species. Each square and size corresponds to the size of the mean effect and the relative weight of the related study, respectively. The vertical line represents the line of no effect, indicating no significant difference in the standardized mean difference.

Detailed parameters of 89 independent studies for the FCR of omnivorous and herbivorous species are presented in Table S4. As illustrated in Figure 5, the BSCP treatment groups showed a nonsignificantly higher FCR than controls (random effects Hedge’s *g* = 0.50, *p* = 0.2431). Analysis revealed high heterogeneity (I^2^ = 96.7%, *p* = 0) and significant publication bias (*p* = 0.0002, Table 2). Correcting for bias via the Trim and Fill method adjusted the effect size to −0.2566 (Table 2). The results were highly robust to missing studies (fail‐safe *N* = 1856), and no significant influence from moderators (BSCP source, species, and habitat; Table 3) was observed.

As mentioned earlier, the typical crude protein content of BSCP used in aquaculture diets ranges from 50% to 75% on a dry weight basis. These levels are above the optimum dietary levels required by carnivorous, omnivorous, and herbivorous species. Excessive protein intakes divert protein function from tissue maintenance and growth to energy production, which is a wasteful process [15, 50, 70]. Moreover, catabolism of excess proteins leads to accumulation of nitrogenous wastes, such as ammonia in rearing water, which can further stress the reared species [77, 78]. Ammonia is toxic to aquaculture species, and its accumulation in rearing water damages their gills, leading to reduced appetite and feed intake (FI), which altogether result in poor FCR [79–81]. Furthermore, stressed fish usually divert proteins for energy production required to detoxify the built‐up ammonia in their tissues, which further leads to poor FCR [82, 83]. Nonetheless, it is imperative to note that carnivorous species usually exhibit low FI upon replacement of FM with other protein sources due to reduced palatability. As such, this leads to high FCR, which translates into poor feed utilization [44]. For instance, Wu et al. [84] observed that replacement of FM with 40% BSCP (*C. autoethanogenum*) reduced the FI and had a negative effect on the intestinal health of large yellow croaker (*Larimichthys crocea*) as evidenced by reduced levels of digestive enzymes (lipase, 6γ‐glutamyltransferase, and acid phosphatase). Low activity of digestive enzymes results in lower breakdown and assimilation of nutrients, thus lower feed utilization [85, 86]. Similarly, research on largemouth bass (*Micropterus salmoides*) showed a decrease in protease activity when the replacement of FM with CAP in their diet exceeded 28.6% [87].

### 3.4. SR

**Figure 6:**
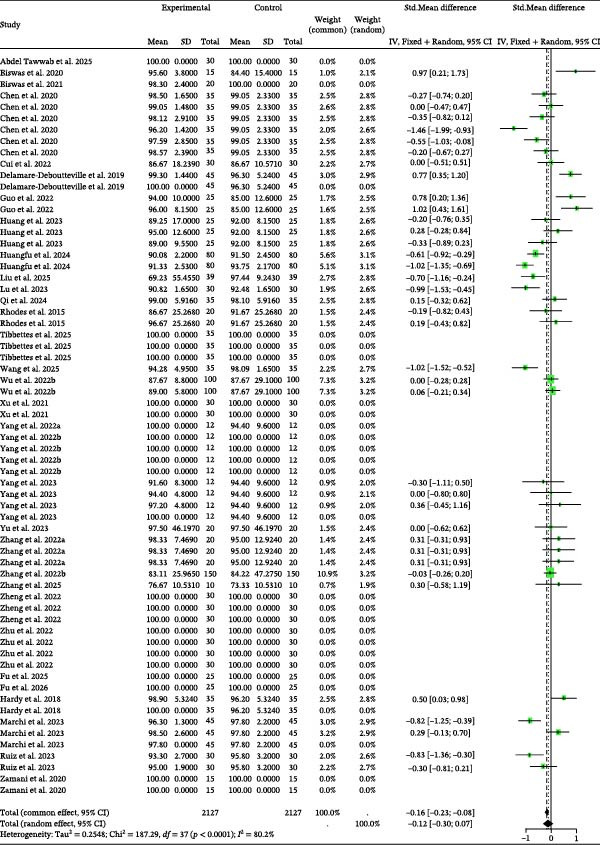
SR forest plot of effect sizes of bacterial single‐cell proteins included in diets of carnivorous aquaculture species. Each square and size corresponds to the size of the mean effect and the relative weight of the related study, respectively. The vertical line represents the line of no effect, indicating no significant difference in the standardized mean difference.

Detailed parameters of 65 independent studies for the SR of carnivorous species are presented in Table S5. The meta‐analysis of SR in carnivorous species (Figure 6) revealed that the SR was nonsignificantly higher in the control group than in the BSCP treatment groups (random effects Hedge’s *g* = −0.12, *p* = 0.2228). The analysis found significant heterogeneity (*I* ^2^ = 80.2%, *p* < 0.0001), but confirmed no publication bias (Table 2) and no significant moderator influence (BSCP source, species, and habitat; Table 3).

**Figure 7:**
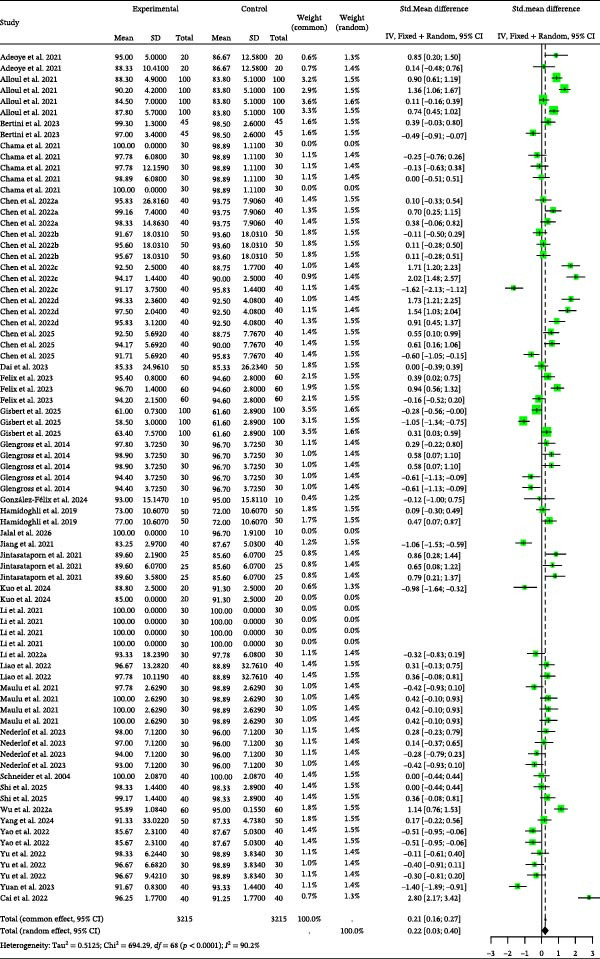
SR forest plot of effect sizes of bacterial single‐cell proteins included in diets of omnivorous and herbivorous aquaculture species. Each square and size corresponds to the size of the mean effect and the relative weight of the related study, respectively. The vertical line represents the line of no effect, indicating no significant difference in the standardized mean difference.

Detailed parameters of 77 independent studies for the SR of omnivorous and herbivorous species are presented in Table S6. The meta‐analysis found a statistically significant positive effect from the dietary inclusion of BSCP (Figure 7). The BSCP treatment groups had significantly higher SR than the control (random effects Hedge’s *g* of 0.22, *p* = 0.0213). This result was observed despite significant heterogeneity across studies (*I* ^2^ = 90.2%, *p* < 0.0001). The analysis confirmed the absence of publication bias (Table 2) and no significant influence from the tested moderators (BSCP source, species, and habitat; Table 3).

The findings demonstrate that BSCPs have a functional immunomodulatory effect on fish and crustaceans under both normal and biotic stress conditions. The bioactive components, including lipopolysaccharides and peptidoglycans, found in the cell walls of bacteria are key [88, 89]. These bioactive compounds act as selective substrates for beneficial indigenous bacteria, which suppress the proliferation of pathogenic microbes through a combination of direct, indirect, and competitive mechanisms, creating a healthier and more resistant intestinal environment [90–92]. These microbes, such as *Lactobacillus*, *Bacillus*, and *Pseudomonas* sp., protect the host by reinforcing the mucosal barrier and enhancing immune function [3]. For instance, fish and crustacean immune cells detect these components, initiating a nonspecific immune response. This reaction successfully stimulates immune‐related enzymes and genes and ultimately enhances the organism’s ability to resist pathogens, hence enhancing the survival of aquaculture species under challenging environments [93, 94]. For instance, Ruiz et al. [95] demonstrated that replacement of FM by BSCP at 50% enhanced the disease resistance of rainbow trout (*Oncorhynchus mykiss*) exposed to *Aeromonas salmonicida* subsp. *salmonicida*. In another study, Jintasataporn et al. [96] reported improved disease resistance against *Vibrio parahaemolyticus* in shrimp (*Penaeus vannamei*) fed 15% BSCP diets. The authors hypothesized that the BSCP meal would interact with Toll‐like receptors (TLRs) in the shrimp’s gut, triggering an innate immune response. This response would, in turn, increase the production of antimicrobial peptides and other enzymes, ultimately boosting the shrimp’s resistance to infection.

### 3.5. Hepatosomatic and Viscerosomatic Indices

**Figure 8:**
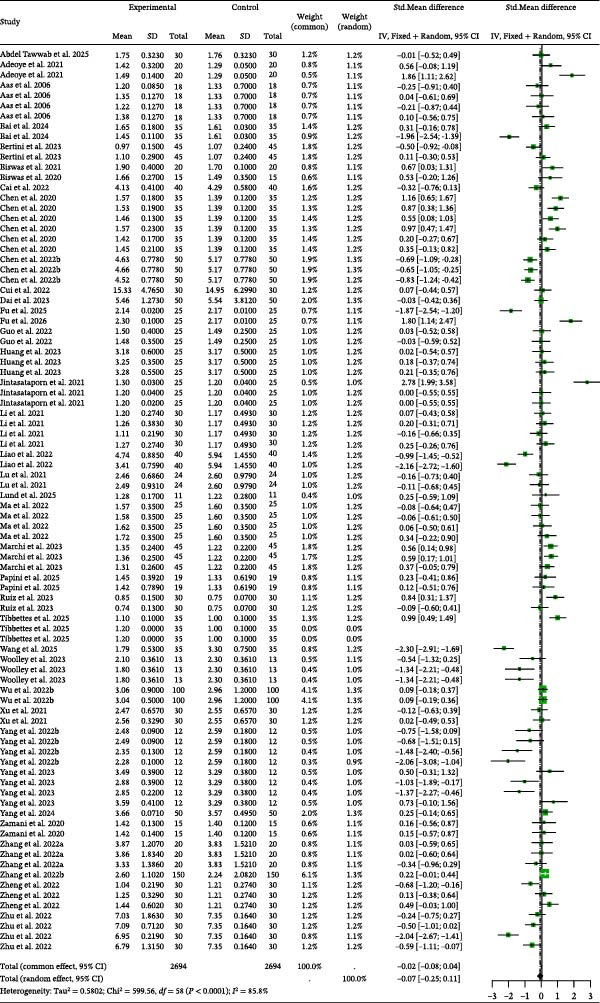
HSI forest plot of effect sizes of bacterial single‐cell proteins included in diets of aquaculture species. Each square and size corresponds to the size of the mean effect and the relative weight of the related study, respectively. The vertical line represents the line of no effect, indicating no significant difference in the standardized mean difference.

Detailed parameters of 88 independent studies for the HSI of aquaculture species are presented in Table S7. As illustrated in Figure 8, the meta‐analysis found a trend suggesting nonsignificantly higher values in the control group compared to the BSCP treatment group (random effects Hedge’s *g* = −0.07, *p* = 0.4771). This result was generated from studies exhibiting significant heterogeneity (*I* ^2^ = 85.8%, *p* < 0.0001). Crucially, the analysis confirmed no evidence of publication bias (Table 2) and found that the selected moderators (BSCP source, species, and habitat) did not significantly explain the variation in the effect sizes (Table 3).

**Figure 9:**
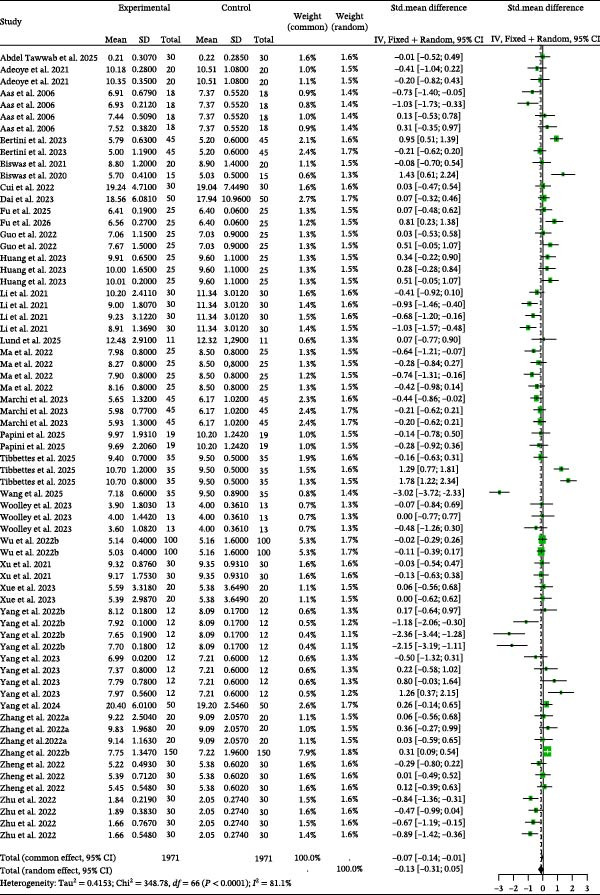
VSI forest plot of effect sizes of bacterial single‐cell proteins included in diets of carnivorous aquaculture species. Each square and size corresponds to the size of the mean effect and the relative weight of the related study, respectively. The vertical line represents the line of no effect, indicating no significant difference in the standardized mean difference.

For VSI, detailed parameters of 67 independent studies are presented in Table S8. Data on VSI (Figure 9) suggested that control groups had nonsignificantly higher values than BSCP treatment groups (random effects *g* = −0.13, *p* = 0.1431). The analysis detected significant between‐study heterogeneity (*I* ^2^ = 81.1%, *p* < 0.0001) and also identified publication bias via Egger’s regression (*p* = 0.0358, Table 2). Correcting for bias using the trim and fill method resulted in an adjusted effect size of 0.1144. Results of the classic fail safe *N* indicated that the missing studies that the number of missing studies that would bring the *p*‐value to 0.05, α is 48. Crucially, the analysis indicated that the BSCP source and the habitat had a significant moderating influence on the VSI effect sizes (Table 3).

As a key indicator of the hepatopancreas condition, the HSI (liver weight relative to body weight) shows a varied response to dietary BSCP. Depending on the aquaculture species and inclusion level, BSCP may have a neutral effect or cause a decrease or increase in HSI. These variations can be viewed either as a positive outcome, signaling improved health and a potential reduction in liver fat accumulation, especially when FM is substituted with other protein sources or as a negative outcome, indicating stress and high metabolic demands [97]. The results of our meta‐analysis revealed that the dietary inclusion of BSCP has a neutral effect on the HSI of several aquaculture species, and thus, a safe alternative protein source in aquaculture nutrition. The inclusion of BSCPs as a FM substitute has been associated with a significant reduction in the HSI in *Litopenaeus vannamei* [38, 98]. This reduction is widely interpreted as a biomarker for enhanced hepatopancreatic health, reflecting optimized lipid metabolism and a decrease in hepatic lipid sequestration, both of which correspond to superior growth performance. These physiological improvements can be explained through the functional “gut–liver axis,” wherein alterations in intestinal microbiota directly modulate hepatic metabolic activity. BSCPs serve as both substrates and modulators for the gut microbiome, fostering the proliferation of beneficial commensal bacteria while concurrently inhibiting the colonization of opportunistic pathogens that might otherwise trigger chronic, low‐grade intestinal inflammation [3]. Moreover, the microbial fermentation of BSCP components produces short‐chain fatty acids (SCFAs), which are absorbed across the intestinal epithelium and transported via the portal circulation to the liver [99]. These metabolites are instrumental in the regulation of hepatic lipid metabolism and insulin sensitivity [100]. By promoting a more favorable microbial composition, BSCPs mitigate the risk of hepatic steatosis, a frequent consequence of suboptimal or unbalanced dietary formulations.

The VSI is the ratio of the total visceral weight to body weight, often indicating variation in the size of the digestive tract, liver, and fat storage. Since the viscera includes adipose tissue, an increase in VSI can be associated with increased visceral fat deposition due to the dietary composition, which is a key consideration when formulating alternative feeds [101]. The results of our meta‐analysis revealed no statistically significant differences between the control and BSCP treatment groups, indicating their safety in aquaculture nutrition. However, our meta‐regression analysis revealed the influence of bacterial species (BSCP source) and habitat on the VSI. Certain bacterial species can increase lipid biosynthesis and absorption, whereas others can trigger the expression of certain genes related to lipid metabolism, hence, promoting lipid deposition in tissues [102, 103]. Lipid deposition and storage are vital for fish, not only offering a concentrated energy reserve for essential life functions like reproduction and enduring starvation stress but also playing a key role in building cell membranes and creating other important biological molecules [104, 105]. Depending on the inclusion level, dietary supplementation of BSCP has been shown to enhance the whole body crude lipid content of several aquaculture species [31, 33, 38, 98].

### 3.6. Biochemical Responses (ALT, AST, SOD, and CAT)

Detailed parameters of 49 independent studies for the alanine transaminase activity (ALT) of aquaculture species are presented in Table S9. Data on ALT activity (Figure S1) initially showed that control groups had a higher enzyme level than BSCP–fed groups (random effects Hedge’s *g* = −0.78, *p* = 0.1116), though this was statistically nonsignificant. Significant heterogeneity (*I* ^2^ = 95%, *p* < 0.01) and publication bias (*p* < 0.01) were detected. Correcting for the bias using the trim and fill method dramatically adjusted the estimated effect size from −0.78 to 0.0513 (Table 2). The classic fail‐safe N of 48 confirmed the stability of the result after correction, and no significant moderator influence was found (Table 3).

Regarding AST activity, detailed parameters of 49 independent studies for the AST activity of aquaculture species are presented in Table S10. The meta‐analysis indicated a trend toward nonsignificantly higher values in the BSCP treatment groups than the controls (random effects Hedge’s *g* = 0.08, *p* = 0.1116), as shown in Figure S2. This finding was drawn from studies exhibiting high between‐study heterogeneity (*I* ^2^ = 93%, *p* < 0.01). Importantly, the analysis showed no evidence of publication bias (Table 2) or any significant explanatory influence from the tested moderators (BSCP source, species, and habitat; Table 3).

Both ALT and AST serve as essential biochemical indicators of hepatic integrity. While these enzymes are primarily involved in intermediate amino acid metabolism, their extracellular release into the blood is significantly upregulated during hepatocellular injury or dysfunction [106, 107]. Such elevations are frequently caused by environmental stressors, including suboptimal salinity and temperature, as well as nutritional imbalances. Recent evidence suggests that the dietary inclusion of BSCPs may exert a modulatory effect on these transaminases [14, 108–110]. For instance, Chen et al. [110] observed that replacing 50% of FM with a composite SCP (*Chlorella vulgaris* and *C. autoethanogenum*) enhanced the hepatic health of *Micropterus salmoides*, as evidenced by a marked reduction in AST and ALT activities. Similarly, Zheng et al. [111] demonstrated that the substitution of FM with 30% methanotroph bacteria meal (*M. capsulatus*) yielded significantly lower ALT and AST levels in juvenile *Scophthalmus maximus L*. compared to those fed conventional plant‐based diets, indicating a superior hepatic safety profile. The hepatoprotective properties of BSCPs can be attributed to several synergistic factors. BSCPs contain nucleotides and small peptides that act as hepatoprotective agents [108, 112]. Furthermore, BSCPs lack the saponins and lectins found in SBM, which are known to cause inflammatory responses in the fish liver (steatosis) that lead to enzyme leakage [113].

In farmed aquatic animals, the nonspecific immune response acts as the primary defense against abiotic and biotic factors [4, 52, 90]. Stress causes increased production and accumulation of reactive oxygen species (ROS), which causes DNA damage, protein carbonylation, and lipid peroxidation [114–116]. To counteract this oxidative stress, the fish rely on antioxidant enzymes like SOD and CAT. For example, SOD performs a crucial protective step by converting the harmful O_2_^−^ (superoxide) radical into the less reactive H_2_O_2_ (hydrogen peroxide). CAT then catalyzes the conversion of H_2_O_2_ to water (H_2_O) and oxygen (O_2_). Overpowering these antioxidant processes often leads to the accumulation of malondialdehyde (MDA), which is an indicator of cellular membrane damage [117]. The dietary inclusion of BSCP in aquaculture diets has been shown to modulate antioxidant enzymatic activity, a phenomenon that is largely contingent upon the specific inclusion levels [55, 96, 98, 118]. This bioactive potential is primarily attributed to the high digestibility of BSCP, which yields a high concentration of low molecular weight peptides [15]. These peptides function both as direct radical scavengers and as molecular ligands capable of activating key signaling pathways, such as the Nrf2 (nuclear factor erythroid 2‐related factor 2) pathway, which regulates the transcription of antioxidant enzymes [119]. Furthermore, the presence of bacterial cell wall constituents, including peptidoglycans and specific β‐glucans, serves as a source of mild immunostimulants. This induces a hormetic effect, wherein a controlled biological stressor triggers a robust protective response. This mechanism effectively elevates the baseline activity of essential enzymes, such as SOD and CAT, thereby enhancing the organism’s oxidative defense without causing systemic inflammation [120].

Table S11 summarizes the detailed parameters of 42 independent studies for the SOD activity of aquaculture species. The meta‐analysis of SOD activity (Figure S3) suggested that BSCP inclusion led to a nonsignificantly higher enzyme activity (random effects Hedge’s *g* = 0.57, *p* = 0.0905). The analysis revealed high heterogeneity (*I* ^2^ = 96%, *p* < 0.01), no publication bias (Table 2), but confirmed a significant moderating influence of the BSCP source and the aquaculture species on the effect size (Table 3).

Table S12 summarizes the detailed parameters of 25 independent studies for the CAT activity of aquaculture species. Data on CAT activity (Figure S4) suggested a strong positive trend in BSCP‐fed groups, but the large initial effect size (random effects Hedge’s *g* = 13.73) was heavily influenced by publication bias (*p* < 0.03, Table 2) and masked by extreme heterogeneity (I^2^ = 98%, *p* < 0.01). Following the trim and fill correction (adding three studies [*n* = 3]), the estimated effect size was adjusted to a much smaller value of 0.2904 (Table 2). The classic fail‐safe *N* of 176 confirmed the stability of the result after correction. Influence diagnostics identified Gisbert et al. [121] as an influential case. A sensitivity analysis was conducted; excluding this study reduced the pooled effect sizes from *g* = 13.73 to *g* = 0.2961 and reduced the *I* ^2^ from 98% to 94.7%. Further analysis confirmed that the BSCP source and aquaculture species were significant moderators of the CAT effect (Table 3).

## 4. Conclusion

This meta‐analysis study explored the influence of the inclusion of BSCPs in aquaculture diets on the growth, physiological homeostasis, and antioxidant capacity of several aquaculture species. The results confirmed that replacement of FM or SBMs with BSCPs improves the growth performance (SGR and survival), feed utilization (FCR), physiological homeostasis (somatic indices and liver health), and antioxidant capacity (SOD, and CAT activities) in aquaculture species under different rearing conditions. We observed very high heterogeneity in our study, and this could be attributed to several factors, such as aquaculture species, microbial species used in the formulation of the aquaculture feed, and rearing conditions. Nonetheless, the overall effect sizes indicated similar responses in growth, antioxidant capacity, and physiological homeostasis of carnivorous, omnivorous, and/or herbivorous species to BSCPs, as indicated by nonstatistically significant differences in the measured parameters between the control and BSCP treatment groups. However, care should be taken during feed formulation since higher inclusion levels of BSCP tend to lower the growth performance of aquaculture species. Overall, depending on the inclusion level, BSCPs are safe and viable alternative proteins for FM or SBMs.

## Author Contributions

Muziri Mugwanya participated in the conceptualization and conducted the data analysis. Fahad Kimera and Muziri Mugwanya participated in research design and data collection. Fahad Kimera, Hani Sewilam, and Muziri Mugwanya participated in paper writing, reviewing, and editing.

## Funding

No funding was received for this study. Open Access funding enabled and organized by Projekt DEAL.

## Disclosure

All authors have reviewed the manuscript.

## Ethics Statement

The authors have nothing to report.

## Consent

The authors have nothing to report.

## Conflicts of Interest

The authors declare no conflicts of interest.

## Supporting Information

Additional supporting information can be found online in the Supporting Information section.

## Supporting information


**Supporting Information** Table S1: Detailed parameters of 74 independent studies for the specific growth (SGR) of carnivorous species. This table includes the author names and year of publication, sample size, mean, and standard deviation of experimental and control groups, respectively. Table S2: Detailed parameters of 84 independent studies for the SGR of omnivorous and herbivorous species. This table includes the author names and year of publication, sample size, mean, and standard deviation of experimental and control groups, respectively. Table S3: Detailed parameters of 75 independent studies for the feed conversion ratio (FCR) of carnivorous species. This table includes the author names and year of publication, sample size, mean, and standard deviation of experimental and control groups, respectively. Table S4: Detailed parameters of 89 independent studies for the FCR of omnivorous and herbivorous species. This table includes the author names and year of publication, sample size, mean, and standard deviation of experimental and control groups, respectively. Table S5: Detailed parameters of 65 independent studies for the SR of carnivorous species. This table includes the author names and year of publication, sample size, mean, and standard deviation of experimental and control groups, respectively. Table S6: Detailed parameters of 77 independent studies for the SR of omnivorous and herbivorous species. This table includes the author names and year of publication, sample size, mean, and standard deviation of experimental and control groups, respectively. Table S7: Detailed parameters of 88 independent studies for the hepatosomatic index (HSI) of aquaculture species. This table includes the author names and year of publication, sample size, mean, and standard deviation of experimental and control groups, respectively. Table S8: Detailed parameters of 67 independent studies for the viscerosomatic index (VSI) of aquaculture species. This table includes the author names and year of publication, sample size, mean, and standard deviation of experimental and control groups, respectively. Table S9: Detailed parameters of 49 independent studies for the alanine transaminase activity (ALT) of aquaculture species. This table includes the author names and year of publication, sample size, mean, and standard deviation of experimental and control groups, respectively. Table S10: Detailed parameters of 49 independent studies for the aspartate aminotransferase (AST) activity of aquaculture species. This table includes the author names and year of publication, sample size, mean, and standard deviation of experimental and control groups, respectively. Table S11: Detailed parameters of 42 independent studies for the superoxide dismutase (SOD) activity of aquaculture species. This table includes the author names and year of publication, sample size, mean, and standard deviation of experimental and control groups, respectively. Table S12: Detailed parameters of 25 independent studies for the catalase (CAT) activity of aquaculture species. This table includes the author names and year of publication, sample size, mean, and standard deviation of experimental and control groups, respectively. Figure S1: ALT forest plot of effect sizes of bacterial single‐cell proteins included in the diets of aquaculture species. Figure S2: AST forest plot of effect sizes of bacterial single‐cell proteins included in the diets of aquaculture species. Figure S3: SOD forest plot of effect sizes of bacterial single‐cell proteins included in the diets of aquaculture species. Figure S4: CAT forest plot of effect sizes of bacterial single‐cell proteins included in the diets of aquaculture species.

## Data Availability

The datasets generated during and/or analyzed during the current study are available from the corresponding author upon reasonable request.
