# Supplementary material for: Bacterial Single‐Cell Proteins as Sustainable Aquafeeds: A Meta‐Analysis of Growth, Physiological Homeostasis, and Antioxidant Capacity
Source: Aquac Nutr. 2026 Apr 25;2026:4548847. doi: 10.1155/anu/4548847 (PMC13110275; doi:10.1155/anu/4548847)
Supplement: Supplementary file 1 — Supporting Information Table S1: Detailed parameters of 74 independent studies for the specific growth (SGR) of carnivorous species. This table includes the author names and year of publication, sample size, mean, and standard deviation of experimental and control groups, respectively. Table S2: Detailed parameters of 84 independent studies for the SGR of omnivorous and herbivorous species. This table includes the author names and year of publication, sample size, mean, and standard deviation of experimental and control groups, respectively. Table S3: Detailed parameters of 75 independent studies for the feed conversion ratio (FCR) of carnivorous species. This table includes the author names and year of publication, sample size, mean, and standard deviation of experimental and control groups, respectively. Table S4: Detailed parameters of 89 independent studies for the FCR of omnivorous and herbivorous species. This table includes the author names and year of publication, sample size, mean, and standard deviation of experimental and control groups, respectively. Table S5: Detailed parameters of 65 independent studies for the SR of carnivorous species. This table includes the author names and year of publication, sample size, mean, and standard deviation of experimental and control groups, respectively. Table S6: Detailed parameters of 77 independent studies for the SR of omnivorous and herbivorous species. This table includes the author names and year of publication, sample size, mean, and standard deviation of experimental and control groups, respectively. Table S7: Detailed parameters of 88 independent studies for the hepatosomatic index (HSI) of aquaculture species. This table includes the author names and year of publication, sample size, mean, and standard deviation of experimental and control groups, respectively. Table S8: Detailed parameters of 67 independent studies for the viscerosomatic index (VSI) of aquaculture species. This table includes the author na [file ANU-2026-4548847-s001.zip › Supplementary Tables.docx]

**Supplementary material**

**Supplementary Table 1**. Detailed parameters of 74 independent studies for the specific growth rate (SGR) of carnivorous species

| Author | n.e | Mean.e | Sd.e | n.c | Mean.c | Sd.c |
| --- | --- | --- | --- | --- | --- | --- |
| Aas et al. [1] | 18 | 1.37 | 0.382 | 18 | 1.38 | 0.45 |
| Aas et al. [1] | 18 | 1.49 | 0.233 | 18 | 1.38 | 0.45 |
| Aas et al. [1] | 18 | 1.59 | 0.42 | 18 | 1.38 | 0.45 |
| Aas et al. [1] | 18 | 1.57 | 0.233 | 18 | 1.38 | 0.45 |
| Abdel Tawwab et al. [2] | 30 | 2.52 | 0.69 | 30 | 2.5 | 0.389 |
| Biswas et al. [3] | 15 | 1.47 | 0.03 | 15 | 1.55 | 0.13 |
| Biswas et al. [4] | 20 | 1.6 | 0.1 | 20 | 1.8 | 0.1 |
| Carvalho et al. [5] | 25 | 0.82 | 0.03 | 25 | 0.81 | 0.02 |
| Carvalho et al. [5] | 25 | 0.8 | 0.03 | 25 | 0.81 | 0.02 |
| Carvalho et al. [6] | 45 | 0.95 | 0.04 | 45 | 1.09 | 0.08 |
| Carvalho et al. [6] | 45 | 0.89 | 0.01 | 45 | 0.97 | 0.02 |
| Chen et al. [7] | 35 | 3.1 | 0.07 | 35 | 3.09 | 0.06 |
| Chen et al. [7] | 35 | 3.09 | 0.05 | 35 | 3.09 | 0.06 |
| Chen et al. [7] | 35 | 3.12 | 0.06 | 35 | 3.09 | 0.06 |
| Chen et al. [7] | 35 | 3.11 | 0.07 | 35 | 3.09 | 0.06 |
| Chen et al. [7] | 35 | 3.08 | 0.03 | 35 | 3.09 | 0.06 |
| Chen et al. [7] | 35 | 3.03 | 0.03 | 35 | 3.09 | 0.06 |
| Cui et al. [8] | 30 | 1.47 | 0.11 | 30 | 1.42 | 0.164 |
| Delamare-Deboutteville et al. [9] | 45 | 3.8 | 0.2 | 45 | 4.2 | 0.3 |
| Delamare-Deboutteville et al. [9] | 45 | 3.7 | 0.1 | 45 | 4.2 | 0.3 |
| Guo et al. [10] | 25 | 1.56 | 0.23 | 25 | 1.56 | 0.27 |
| Guo et al. [10] | 25 | 1.64 | 0.305 | 25 | 1.56 | 0.27 |
| Huang et al. [11] | 25 | 2.98 | 0.05 | 25 | 2.98 | 0.15 |
| Huang et al. [11] | 25 | 2.93 | 0.15 | 25 | 2.98 | 0.15 |
| Huang et al. [11] | 25 | 2.91 | 0.05 | 25 | 2.98 | 0.15 |
| Kiessling et al. [12] | 30 | 0.73 | 0.05 | 30 | 0.75 | 0.08 |
| Kiessling et al. [12] | 30 | 0.69 | 0.05 | 30 | 0.75 | 0.08 |
| Kiessling et al. [12] | 30 | 0.71 | 0.05 | 30 | 0.75 | 0.08 |
| Lu et al. [13] | 24 | 2.62 | 0.245 | 24 | 2.45 | 0.392 |
| Lu et al. [13] | 24 | 2.52 | 0.049 | 24 | 2.45 | 0.392 |
| Lu et al. [14] | 30 | 0.74 | 0.03 | 30 | 0.75 | 0.05 |
| Ma et al. [15] | 25 | 2.11 | 0.15 | 25 | 2.2 | 0.15 |
| Ma et al. [15] | 25 | 2.17 | 0.15 | 25 | 2.2 | 0.15 |
| Ma et al. [15] | 25 | 2.13 | 0.15 | 25 | 2.2 | 0.15 |
| Ma et al. [15] | 25 | 2.08 | 0.15 | 25 | 2.2 | 0.15 |
| Pilmer et al. [16] | 13 | 2.2 | 0.361 | 13 | 2.7 | 0.361 |
| Pilmer et al. [16] | 13 | 2.6 | 0.361 | 13 | 2.7 | 0.361 |
| Pilmer et al. [16] | 13 | 2.2 | 0.361 | 13 | 2.7 | 0.361 |
| Rizwan et al. [17] | 10 | 0.22 | 0.04 | 10 | 0.69 | 0.04 |
| Tibbettes et al. [18] | 35 | 1.4 | 0.1 | 35 | 1.4 | 0.1 |
| Tibbettes et al. [18] | 35 | 1.2 | 0.1 | 35 | 1.4 | 0.1 |
| Tibbettes et al. [18] | 35 | 1.1 | 0.1 | 35 | 1.4 | 0.1 |
| Woolley et al. [19] | 13 | 2 | 0.361 | 13 | 1.8 | 0.361 |
| Woolley et al. [19] | 13 | 2 | 0.361 | 13 | 1.8 | 0.361 |
| Woolley et al. [19] | 13 | 2.1 | 0.361 | 13 | 1.8 | 0.361 |
| Xu et al. [20] | 30 | 2.57 | 0.055 | 30 | 2.52 | 0.055 |
| Xu et al. [20] | 30 | 2.72 | 0.164 | 30 | 2.52 | 0.055 |
| Yang et al. [21] | 12 | 1.27 | 0.06 | 12 | 1.34 | 0.06 |
| Yu et al. [22] | 20 | 2.21 | 0.447 | 20 | 2.06 | 0.447 |
| Zhang et al. [23] | 20 | 2.71 | 0.224 | 20 | 2.72 | 0.358 |
| Zhang et al. [23] | 20 | 2.68 | 0.134 | 20 | 2.72 | 0.358 |
| Zhang et al. [23] | 20 | 2.59 | 0.134 | 20 | 2.72 | 0.358 |
| Zhang et al. [24] | 10 | 1.29 | 0.032 | 10 | 1.2 | 0.19 |
| Zheng et al. [25] | 30 | 2.76 | 0.438 | 30 | 2.87 | 0.383 |
| Zheng et al. [25] | 30 | 2.73 | 0.164 | 30 | 2.87 | 0.383 |
| Zheng et al. [25] | 30 | 2.56 | 0.274 | 30 | 2.87 | 0.383 |
| Zhu et al. [26] | 30 | 1.81 | 0.548 | 30 | 1.87 | 0.219 |
| Zhu et al. [26] | 30 | 1.89 | 0.548 | 30 | 1.87 | 0.219 |
| Zhu et al. [26] | 30 | 1.9 | 0.493 | 30 | 1.87 | 0.219 |
| Zhu et al. [26] | 30 | 1.94 | 0.274 | 30 | 1.87 | 0.219 |
| Hardy et al. [27] | 35 | 3.21 | 0.177 | 35 | 3.24 | 0.059 |
| Hardy et al. [27] | 35 | 3.16 | 0.118 | 35 | 3.24 | 0.059 |
| Lund et al. [28] | 11 | 2.57 | 0.01 | 11 | 2.59 | 0.02 |
| Papini et al. [29] | 19 | 2.46 | 1.255 | 19 | 2.38 | 0.362 |
| Papini et al. [29] | 19 | 2.2 | 0.501 | 19 | 2.38 | 0.362 |
| Ruiz et al. [30] | 30 | 3.45 | 0.02 | 30 | 3.41 | 0.04 |
| Ruiz et al. [30] | 30 | 3.47 | 0.04 | 30 | 3.41 | 0.04 |
| Zamani et al. [31] | 15 | 2.9 | 0.06 | 15 | 2.74 | 0.13 |
| Zamani et al. [31] | 15 | 3.15 | 0.02 | 15 | 2.74 | 0.13 |
| Marchi et al. [32] | 45 | 0.98 | 0.01 | 45 | 0.98 | 0.02 |
| Marchi et al. [32] | 45 | 0.96 | 0.01 | 45 | 0.98 | 0.02 |
| Marchi et al. [32] | 45 | 0.97 | 0.03 | 45 | 0.98 | 0.02 |
| Fu et al. [33] | 25 | 2.27 | 0.13 | 25 | 2.18 | 0.1 |

**Supplementary Table 2**. Detailed parameters of 84 independent studies for the specific growth rate (SGR) of omnivorous and herbivorous species

| Author | n.e | Mean.e | Sd.e | n.c | Mean.c | Sd.c |
| --- | --- | --- | --- | --- | --- | --- |
| Adeoye et al. [34] | 20 | 2.3 | 0.08 | 20 | 2.4 | 0.04 |
| Adeoye et al. [34] | 20 | 2.28 | 0.07 | 20 | 2.4 | 0.04 |
| Alloul et al. [35] | 100 | 13.2 | 0.3 | 100 | 12.6 | 0.5 |
| Alloul et al. [35] | 100 | 13.2 | 0.3 | 100 | 12.6 | 0.5 |
| Alloul et al. [35] | 100 | 12.8 | 0.2 | 100 | 12.6 | 0.5 |
| Alloul et al. [35] | 100 | 13.4 | 0.6 | 100 | 12.6 | 0.5 |
| Bertini et al. [36] | 45 | 0.62 | 0.07 | 45 | 0.88 | 0.04 |
| Bertini et al. [36] | 45 | 0.41 | 0.02 | 45 | 0.88 | 0.04 |
| Cai et al. [37] | 50 | 1.3 | 0.08 | 50 | 1.5 | 0.07 |
| Chama et al. [38] | 30 | 5.41 | 0.111 | 30 | 5.58 | 0.383 |
| Chama et al. [38] | 30 | 5.36 | 0.111 | 30 | 5.58 | 0.383 |
| Chama et al. [38] | 30 | 5.37 | 0.164 | 30 | 5.58 | 0.383 |
| Chama et al. [38] | 30 | 5.34 | 0.329 | 30 | 5.58 | 0.383 |
| Chama et al. [38] | 30 | 5.43 | 0.055 | 30 | 5.58 | 0.383 |
| Dai et al. [39] | 50 | 2.01 | 0.495 | 50 | 1.83 | 0.071 |
| Fan et al. [40] | 25 | 1.2 | 0 | 25 | 1.39 | 0.03 |
| Fan et al. [40] | 25 | 1.21 | 0.13 | 25 | 1.48 | 0.03 |
| Felix et al. [41] | 60 | 2.83 | 0.09 | 60 | 2.77 | 0.05 |
| Felix et al. [41] | 60 | 2.96 | 0.04 | 60 | 2.77 | 0.05 |
| Felix et al. [41] | 60 | 2.98 | 0.03 | 60 | 2.77 | 0.05 |
| Gisbert et al. [42] | 100 | 3.35 | 0.03 | 100 | 3.4 | 0.02 |
| Gisbert et al. [42] | 100 | 3.35 | 0.06 | 100 | 3.4 | 0.02 |
| Gisbert et al. [42] | 100 | 3.26 | 0.04 | 100 | 3.4 | 0.02 |
| Glengross et al. [43] | 30 | 0.86 | 0.111 | 30 | 0.58 | 0.111 |
| Glengross et al. [43] | 30 | 0.93 | 0.111 | 30 | 0.58 | 0.111 |
| Glengross et al. [43] | 30 | 0.8 | 0.111 | 30 | 0.58 | 0.111 |
| Glengross et al. [43] | 30 | 1.03 | 0.111 | 30 | 0.58 | 0.111 |
| Glengross et al. [43] | 30 | 1.06 | 0.111 | 30 | 0.58 | 0.111 |
| González-Félix et al. [44] | 10 | 12.33 | 0.949 | 10 | 12.92 | 0.285 |
| Hamidoghli et al. [45] | 50 | 3.65 | 0.283 | 50 | 3.63 | 0.283 |
| Hamidoghli et al. [45] | 50 | 3.49 | 0.283 | 50 | 3.63 | 0.283 |
| Jalal et al. [46] | 10 | 0.49 | 0.09 | 10 | 0.42 | 0.06 |
| Jiang et al. [47] | 40 | 2.6 | 0.11 | 40 | 2.64 | 0.08 |
| Jintasataporn et al. [48] | 25 | 3.44 | 0.24 | 25 | 3.4 | 0.25 |
| Jintasataporn et al. [48] | 25 | 3.45 | 0.2 | 25 | 3.4 | 0.25 |
| Jintasataporn et al. [48] | 25 | 3.61 | 0.28 | 25 | 3.4 | 0.25 |
| Kuo et al. [49] | 20 | 3.13 | 0.15 | 20 | 3.15 | 0.04 |
| Kuo et al. [49] | 20 | 3.07 | 0.06 | 20 | 3.15 | 0.04 |
| Li et al. [50] | 30 | 3.73 | 0.164 | 30 | 3.64 | 0.219 |
| Li et al. [50] | 30 | 3.83 | 0.164 | 30 | 3.64 | 0.219 |
| Li et al. [50] | 30 | 3.91 | 0.111 | 30 | 3.64 | 0.219 |
| Li et al. [50] | 30 | 3.95 | 0.111 | 30 | 3.64 | 0.219 |
| Li et al. [51] | 30 | 2.34 | 0.164 | 30 | 2.33 | 0.383 |
| Liao et al. [52] | 40 | 3.9 | 0.126 | 40 | 3.5 | 0.379 |
| Liao et al. [52] | 40 | 3.74 | 0.759 | 40 | 3.5 | 0.379 |
| Maulu et al. [53] | 30 | 6.15 | 0.329 | 30 | 5.63 | 0.329 |
| Maulu et al. [53] | 30 | 6.17 | 0.329 | 30 | 5.63 | 0.329 |
| Maulu et al. [53] | 30 | 6.22 | 0.329 | 30 | 5.63 | 0.329 |
| Maulu et al. [53] | 30 | 6.28 | 0.329 | 30 | 5.63 | 0.329 |
| Nederlof et al. [54] | 30 | 2.37 | 0.219 | 30 | 2.26 | 0.219 |
| Nederlof et al. [54] | 30 | 2.24 | 0.219 | 30 | 2.26 | 0.219 |
| Nederlof et al. [54] | 30 | 2.3 | 0.219 | 30 | 2.26 | 0.219 |
| Nederlof et al. [54] | 30 | 2.36 | 0.219 | 30 | 2.26 | 0.219 |
| Salini et al. [55] | 24 | 0.82 | 0.294 | 24 | 0.87 | 0.147 |
| Salini et al. [55] | 24 | 0.78 | 0.245 | 24 | 0.87 | 0.147 |
| Schneider et al. [56] | 40 | 1.9 | 0.19 | 40 | 2.1 | 0.19 |
| Shi et al. [57] | 40 | 3.8 | 0 | 40 | 3.85 | 0.05 |
| Shi et al. [57] | 40 | 3.69 | 0.07 | 40 | 3.85 | 0.05 |
| Tlusty et al. [58] | 60 | 2.81 | 0.697 | 60 | 2.92 | 0.387 |
| Tlusty et al. [58] | 60 | 2.64 | 1.084 | 60 | 2.92 | 0.387 |
| Wu et al. [59] | 30 | 1.11 | 0.274 | 30 | 1.71 | 1.15 |
| Wu et al. [59] | 30 | 1.19 | 0.329 | 30 | 2.44 | 0.657 |
| Wu et al. [59] | 30 | 1.55 | 0.712 | 30 | 2.62 | 1.588 |
| Xue et al. [60] | 20 | 0.87 | 0.04 | 20 | 0.83 | 0.112 |
| Xue et al. [60] | 20 | 0.81 | 0.107 | 20 | 0.83 | 0.112 |
| Yu et al. [61] | 30 | 2.69 | 0.11 | 30 | 2.58 | 0.055 |
| Yu et al. [61] | 30 | 2.7 | 0.055 | 30 | 2.58 | 0.055 |
| Yu et al. [61] | 30 | 2.66 | 0.055 | 30 | 2.58 | 0.055 |
| Yuan et al. [62] | 40 | 2.24 | 0.03 | 40 | 1.96 | 0.07 |
| Chen et al. [63] | 40 | 4.27 | 0.19 | 40 | 4.34 | 0.126 |
| Chen et al. [63] | 40 | 4.1 | 0.443 | 40 | 4.34 | 0.126 |
| Chen et al. [63] | 40 | 4.2 | 0.443 | 40 | 4.34 | 0.126 |
| Chen et al. [64] | 50 | 6.42 | 0.636 | 50 | 5.84 | 0.636 |
| Chen et al. [64] | 50 | 6.36 | 0.636 | 50 | 5.84 | 0.636 |
| Chen et al. [64] | 50 | 6.47 | 0.636 | 50 | 5.84 | 0.636 |
| Chen et al. [65] | 40 | 5.3 | 0.03 | 40 | 5.22 | 0.03 |
| Chen et al. [65] | 40 | 5.42 | 0.05 | 40 | 5.24 | 0.03 |
| Chen et al. [65] | 40 | 5.25 | 0.02 | 40 | 5.05 | 0.12 |
| Chen et al. [66] | 40 | 2.78 | 0.03 | 40 | 2.87 | 0.04 |
| Chen et al. [66] | 40 | 2.77 | 0.04 | 40 | 2.87 | 0.04 |
| Chen et al. [66] | 40 | 2.88 | 0.03 | 40 | 2.87 | 0.04 |
| Chen et al. [67] | 40 | 5.3 | 0.177 | 40 | 5.22 | 0.234 |
| Chen et al. [67] | 40 | 5.42 | 0.177 | 40 | 5.24 | 0.234 |

**Supplementary Table 3**. Detailed parameters of 75 independent studies for the feed conversion ratio (FCR) of carnivorous species

| Author | n.e | Mean.e | Sd.e | n.c | Mean.c | Sd.c |
| --- | --- | --- | --- | --- | --- | --- |
| Aas et al. [1] | 18 | 1.36 | 0.042 | 18 | 1.39 | 0.212 |
| Aas et al. [1] | 18 | 1.45 | 0.042 | 18 | 1.39 | 0.212 |
| Aas et al. [1] | 18 | 1.46 | 0.11 | 18 | 1.39 | 0.212 |
| Aas et al. [1] | 18 | 1.55 | 0.148 | 18 | 1.39 | 0.212 |
| Abdel Tawwab et al. [2] | 30 | 1.54 | 0.652 | 30 | 1.59 | 0.394 |
| Bai et al. [68] | 35 | 1.05 | 0.04 | 35 | 1 | 0.06 |
| Bai et al. [68] | 35 | 1.13 | 0.09 | 35 | 1 | 0.06 |
| Berge et al. [69] | 1000 | 1.09 | 0.949 | 1000 | 1.02 | 0.316 |
| Berge et al. [69] | 1000 | 1.09 | 0 | 1000 | 1.11 | 0.316 |
| Berge et al. [69] | 1000 | 1.27 | 0.949 | 1000 | 1.22 | 0.632 |
| Berge et al. [69] | 1000 | 1.13 | 0 | 1000 | 1.12 | 0 |
| Carvalho et al. [5] | 25 | 1.57 | 0.07 | 25 | 1.54 | 0.08 |
| Carvalho et al. [5] | 25 | 1.54 | 0.11 | 25 | 1.54 | 0.08 |
| Carvalho et al. [6] | 45 | 1.6 | 0.04 | 45 | 1.45 | 0.16 |
| Carvalho et al. [6] | 45 | 1.76 | 0.05 | 45 | 1.65 | 0.04 |
| Cui et al. [8] | 30 | 1.43 | 0.11 | 30 | 1.52 | 0.274 |
| Guo et al. [10] | 25 | 1.16 | 0.2 | 25 | 1.22 | 0.35 |
| Guo et al. [10] | 25 | 1.13 | 0.1 | 25 | 1.22 | 0.35 |
| Huang et al. [11] | 25 | 0.8 | 0 | 25 | 0.8 | 0.05 |
| Huang et al. [11] | 25 | 0.81 | 0.05 | 25 | 0.8 | 0.05 |
| Huang et al. [11] | 25 | 0.81 | 0.05 | 25 | 0.8 | 0.05 |
| Lu et al. [13] | 24 | 0.73 | 0.147 | 24 | 0.76 | 0.147 |
| Lu et al. [13] | 24 | 0.89 | 0.098 | 24 | 0.76 | 0.147 |
| Ma et al. [15] | 25 | 0.89 | 0.05 | 25 | 0.87 | 0.05 |
| Ma et al. [15] | 25 | 0.89 | 0.05 | 25 | 0.87 | 0.05 |
| Ma et al. [15] | 25 | 0.88 | 0.05 | 25 | 0.87 | 0.05 |
| Ma et al. [15] | 25 | 0.89 | 0.05 | 25 | 0.87 | 0.05 |
| Pilmer et al. [16] | 13 | 1.07 | 0.072 | 13 | 1.14 | 0 |
| Pilmer et al. [16] | 13 | 1.08 | 0.036 | 13 | 1.14 | 0 |
| Pilmer et al. [16] | 13 | 1.07 | 0.253 | 13 | 1.14 | 0 |
| Rhodes et al. [70] | 20 | 1.99 | 0.631 | 20 | 2.1 | 0.631 |
| Rhodes et al. [70] | 20 | 2.09 | 0.631 | 20 | 2.1 | 0.631 |
| Rizwan et al. [17] | 10 | 2.12 | 0.65 | 10 | 1.27 | 0.06 |
| Woolley et al. [19] | 35 | 0.97 | 0.08 | 35 | 0.72 | 0.01 |
| Woolley et al. [19] | 13 | 1.02 | 0.036 | 13 | 0.96 | 0.036 |
| Woolley et al. [19] | 13 | 1.1 | 0.072 | 13 | 0.96 | 0.036 |
| Woolley et al. [19] | 13 | 1.13 | 0.036 | 13 | 0.96 | 0.036 |
| Xu et al. [20] | 30 | 1.23 | 0.11 | 30 | 1.27 | 0.164 |
| Xu et al. [20] | 30 | 1.22 | 0.055 | 30 | 1.27 | 0.164 |
| Yang et al. [21] | 12 | 1.33 | 0.09 | 12 | 1.28 | 0.04 |
| Yang et al. [71] | 12 | 1.04 | 0.04 | 12 | 1.06 | 0.02 |
| Yang et al. [71] | 12 | 1 | 0.01 | 12 | 1.06 | 0.02 |
| Yang et al. [71] | 12 | 1.1 | 0.03 | 12 | 1.06 | 0.02 |
| Yang et al. [71] | 12 | 1.13 | 0 | 12 | 1.06 | 0.02 |
| Yang et al. [72] | 12 | 1.26 | 0.11 | 12 | 1.28 | 0.04 |
| Yang et al. [72] | 12 | 1.29 | 0.07 | 12 | 1.28 | 0.04 |
| Yang et al. [72] | 12 | 1.33 | 0.09 | 12 | 1.28 | 0.04 |
| Yang et al. [72] | 12 | 1.29 | 0.09 | 12 | 1.28 | 0.04 |
| Yu et al. [22] | 20 | 0.98 | 0.402 | 20 | 1 | 0.402 |
| Zhang et al. [23] | 20 | 1.13 | 0.134 | 20 | 1.16 | 0.179 |
| Zhang et al. [23] | 20 | 1.18 | 0.179 | 20 | 1.16 | 0.179 |
| Zhang et al. [23] | 20 | 1.24 | 0.134 | 20 | 1.16 | 0.179 |
| Zheng et al. [25] | 30 | 1.59 | 0.164 | 30 | 1.52 | 0.219 |
| Zheng et al. [25] | 30 | 1.52 | 0.164 | 30 | 1.52 | 0.219 |
| Zheng et al. [25] | 30 | 1.51 | 0.219 | 30 | 1.52 | 0.219 |
| Zhu et al. [26] | 30 | 0.91 | 0.11 | 30 | 0.95 | 0.164 |
| Zhu et al. [26] | 30 | 0.88 | 0.164 | 30 | 0.95 | 0.164 |
| Zhu et al. [26] | 30 | 0.87 | 0.219 | 30 | 0.95 | 0.164 |
| Zhu et al. [26] | 30 | 0.88 | 0.164 | 30 | 0.95 | 0.164 |
| Zamani et al. [31] | 15 | 0.89 | 0.04 | 15 | 0.96 | 0.01 |
| Zamani et al. [31] | 15 | 0.76 | 0.01 | 15 | 0.96 | 0.01 |
| Hardy et al. [27] | 35 | 0.82 | 0.059 | 35 | 0.85 | 0.059 |
| Hardy et al. [27] | 35 | 0.84 | 0 | 35 | 0.85 | 0.059 |
| Lund et al. [28] | 11 | 0.77 | 0 | 11 | 0.77 | 0.01 |
| Marchi et al. [32] | 45 | 1.28 | 0.05 | 45 | 1.29 | 0.04 |
| Marchi et al. [32] | 45 | 1.35 | 0.01 | 45 | 1.29 | 0.04 |
| Marchi et al. [32] | 45 | 1.35 | 0.05 | 45 | 1.29 | 0.04 |
| Papini et al. [29] | 19 | 1.01 | 0.414 | 19 | 1.1 | 0.558 |
| Papini et al. [29] | 19 | 1.18 | 0.497 | 19 | 1.1 | 0.558 |
| Ruiz et al. [30] | 30 | 0.81 | 0.02 | 30 | 0.81 | 0.01 |
| Ruiz et al. [30] | 30 | 0.79 | 0.02 | 30 | 0.81 | 0.01 |
| Fu et al. [33] | 25 | 1.84 | 0.06 | 25 | 1.89 | 0.12 |
| Fu et al. [33] | 25 | 1.44 | 0.1 | 25 | 1.89 | 0.12 |
| Wu et al. [73] | 100 | 1.62 | 0.1 | 100 | 1.58 | 0.4 |
| Wu et al. [73] | 100 | 1.61 | 0.1 | 100 | 1.58 | 0.4 |

**Supplementary Table 4**. Detailed parameters of 89 independent studies for the feed conversion ratio (FCR) of omnivorous and herbivorous species

| Author | n.e | Mean.e | Sd.e | n.c | Mean.c | Sd.c |
| --- | --- | --- | --- | --- | --- | --- |
| Adeoye et al. [34] | 20 | 1.52 | 0.08 | 20 | 1.55 | 0.06 |
| Adeoye et al. [34] | 20 | 1.59 | 0.03 | 20 | 1.55 | 0.06 |
| Alloul et al. [35] | 100 | 1.4 | 0.1 | 100 | 1.7 | 0.2 |
| Alloul et al. [35] | 100 | 1.3 | 0.1 | 100 | 1.7 | 0.2 |
| Alloul et al. [35] | 100 | 1.7 | 0.2 | 100 | 1.7 | 0.2 |
| Alloul et al. [35] | 100 | 1.5 | 0.1 | 100 | 1.7 | 0.2 |
| Bertini et al. [36] | 45 | 2.69 | 0.36 | 45 | 1.8 | 0.1 |
| Bertini et al. [36] | 45 | 4.8 | 0.12 | 45 | 1.8 | 0.1 |
| Cai et al. [37] | 40 | 1.69 | 0.03 | 40 | 1.75 | 0.04 |
| Cai et al. [74] | 50 | 1.97 | 0.14 | 50 | 1.74 | 0.09 |
| Chama et al. [38] | 30 | 0.86 | 0.055 | 30 | 0.87 | 0.219 |
| Chama et al. [38] | 30 | 0.87 | 0.055 | 30 | 0.87 | 0.219 |
| Chama et al. [38] | 30 | 0.86 | 0.055 | 30 | 0.87 | 0.219 |
| Chama et al. [38] | 30 | 0.88 | 0.164 | 30 | 0.87 | 0.219 |
| Chama et al. [38] | 30 | 0.84 | 0.055 | 30 | 0.87 | 0.219 |
| Chen et al. [63] | 40 | 1.67 | 0.379 | 40 | 1.62 | 0.253 |
| Chen et al. [63] | 40 | 1.7 | 0.126 | 40 | 1.62 | 0.253 |
| Chen et al. [63] | 40 | 1.63 | 0.253 | 40 | 1.62 | 0.253 |
| Chen et al. [64] | 50 | 1.28 | 1.26 | 50 | 1.26 | 0.566 |
| Chen et al. [64] | 50 | 1.34 | 1.26 | 50 | 1.26 | 0.566 |
| Chen et al. [64] | 50 | 1.25 | 1.26 | 50 | 1.26 | 0.566 |
| Chen et al. [65] | 40 | 1.04 | 0.05 | 40 | 1.41 | 0.12 |
| Chen et al. [65] | 40 | 1.98 | 0.09 | 40 | 1.49 | 0.04 |
| Chen et al. [65] | 40 | 3.69 | 0.09 | 40 | 1.39 | 0.1 |
| Chen et al. [66] | 40 | 1.46 | 0.04 | 40 | 1.5 | 0.08 |
| Chen et al. [66] | 40 | 1.48 | 0.02 | 40 | 1.5 | 0.08 |
| Chen et al. [66] | 40 | 1.42 | 0.09 | 40 | 1.5 | 0.08 |
| Chen et al. [67] | 40 | 1.04 | 2.46 | 40 | 1.41 | 0.196 |
| Chen et al. [67] | 40 | 1.98 | 2.46 | 40 | 1.49 | 0.196 |
| Chen et al. [67] | 40 | 3.69 | 2.46 | 40 | 1.39 | 0.196 |
| Dai et al. [39] | 50 | 1.32 | 0.071 | 50 | 1.38 | 0.212 |
| Fan et al. [40] | 25 | 2.29 | 0.13 | 25 | 1.64 | 0.07 |
| Fan et al. [40] | 25 | 2.42 | 0.29 | 25 | 1.84 | 0.05 |
| Felix et al. [41] | 60 | 0.47 | 0.01 | 60 | 0.43 | 0.01 |
| Felix et al. [41] | 60 | 0.52 | 0.03 | 60 | 0.43 | 0.01 |
| Felix et al. [41] | 60 | 0.56 | 0.01 | 60 | 0.43 | 0.01 |
| Gisbert et al. [42] | 100 | 1.68 | 0.27 | 100 | 1.83 | 0.21 |
| Gisbert et al. [42] | 100 | 1.69 | 0.18 | 100 | 1.83 | 0.21 |
| Gisbert et al. [42] | 100 | 1.7 | 0.57 | 100 | 1.83 | 0.21 |
| Glengross et al. [43] | 30 | 4.16 | 0.438 | 30 | 4.74 | 0.438 |
| Glengross et al. [43] | 30 | 3.86 | 0.438 | 30 | 4.74 | 0.438 |
| Glengross et al. [43] | 30 | 3.89 | 0.438 | 30 | 4.74 | 0.438 |
| Glengross et al. [43] | 30 | 3.68 | 0.438 | 30 | 4.74 | 0.438 |
| Glengross et al. [43] | 30 | 3.18 | 0.438 | 30 | 4.74 | 0.438 |
| González-Félix et al. [44] | 10 | 1.26 | 0.158 | 10 | 1.2 | 0.032 |
| Hamidoghli et al. [45] | 50 | 1.56 | 0.354 | 50 | 1.59 | 0.354 |
| Hamidoghli et al. [45] | 50 | 1.8 | 0.354 | 50 | 1.59 | 0.354 |
| Jalal et al. [46] | 10 | 1.77 | 0.33 | 10 | 1.9 | 0.12 |
| Jiang et al. [47] | 40 | 1.21 | 0.01 | 40 | 1.18 | 0.02 |
| Jintasataporn et al. [48] | 25 | 1.21 | 0.13 | 25 | 1.26 | 0.09 |
| Jintasataporn et al. [48] | 25 | 1.21 | 0.05 | 25 | 1.26 | 0.09 |
| Jintasataporn et al. [48] | 25 | 1.15 | 0.15 | 25 | 1.26 | 0.09 |
| Kuo et al. [49] | 20 | 1.24 | 0.06 | 20 | 1.2 | 0.04 |
| Kuo et al. [49] | 20 | 1.27 | 0.02 | 20 | 1.2 | 0.04 |
| Li et al. [50] | 30 | 1.09 | 0.111 | 30 | 1.2 | 0.164 |
| Li et al. [50] | 30 | 1.04 | 0.111 | 30 | 1.2 | 0.164 |
| Li et al. [50] | 30 | 0.97 | 0.055 | 30 | 1.2 | 0.164 |
| Li et al. [50] | 30 | 0.94 | 0.055 | 30 | 1.2 | 0.164 |
| Li et al. [51] | 30 | 1.47 | 0.657 | 30 | 1.72 | 0.164 |
| Liao et al. [52] | 40 | 1.71 | 0.379 | 40 | 2.19 | 0.696 |
| Liao et al. [52] | 40 | 1.86 | 0.19 | 40 | 2.19 | 0.696 |
| Maulu et al. [53] | 30 | 0.99 | 0.219 | 30 | 1.29 | 0.219 |
| Maulu et al. [53] | 30 | 0.96 | 0.219 | 30 | 1.29 | 0.219 |
| Maulu et al. [53] | 30 | 0.97 | 0.219 | 30 | 1.29 | 0.219 |
| Maulu et al. [53] | 30 | 0.94 | 0.219 | 30 | 1.29 | 0.219 |
| Nederlof et al. [54] | 30 | 1.64 | 0.279 | 30 | 1.71 | 0.279 |
| Nederlof et al. [54] | 30 | 1.71 | 0.279 | 30 | 1.71 | 0.279 |
| Nederlof et al. [54] | 30 | 1.68 | 0.279 | 30 | 1.71 | 0.279 |
| Nederlof et al. [54] | 30 | 1.58 | 0.279 | 30 | 1.71 | 0.279 |
| Salini et al. [55] | 24 | 0.93 | 0.441 | 24 | 0.87 | 0.098 |
| Salini et al. [55] | 24 | 0.95 | 0.294 | 24 | 0.87 | 0.098 |
| Schneider et al. [56] | 40 | 1.1 | 0.127 | 40 | 1 | 0.127 |
| Shi et al. [57] | 40 | 1.42 | 0.02 | 40 | 1.38 | 0.07 |
| Shi et al. [57] | 40 | 1.49 | 0.02 | 40 | 1.38 | 0.07 |
| Tlusty et al. [58] | 60 | 1.59 | 0.465 | 60 | 1.7 | 0.93 |
| Tlusty et al. [58] | 60 | 1.95 | 0.387 | 60 | 1.7 | 0.93 |
| Wu et al. 2022a | 60 | 2.48 | 0.31 | 60 | 2.95 | 0.465 |
| Wu et al. [59] | 30 | 2.09 | 0.986 | 30 | 1.7 | 0.876 |
| Wu et al. [59] | 30 | 1.49 | 0.657 | 30 | 1.11 | 0.602 |
| Wu et al. [59] | 30 | 1.45 | 1.041 | 30 | 0.98 | 0.384 |
| Xue et al. [60] | 20 | 1.76 | 0.112 | 20 | 1.83 | 0.295 |
| Xue et al. [60] | 20 | 1.82 | 0.067 | 20 | 1.83 | 0.295 |
| Yang et al. [75] | 50 | 1.51 | 0.495 | 50 | 1.71 | 0.283 |
| Yao et al. [76] | 40 | 1.2 | 0.01 | 40 | 1.18 | 0.04 |
| Yao et al. [76] | 40 | 1.2 | 0.06 | 40 | 1.18 | 0.04 |
| Yu et al. [61] | 30 | 1.15 | 0.11 | 30 | 1.32 | 0.11 |
| Yu et al. [61] | 30 | 1.17 | 0.164 | 30 | 1.32 | 0.11 |
| Yu et al. [61] | 30 | 1.21 | 0.11 | 30 | 1.32 | 0.11 |
| Yuan et al. [62] | 40 | 1.85 | 0.04 | 40 | 2.07 | 0.12 |

**Supplementary Table 5**. Detailed parameters of 65 independent studies for the survival rate (SR) of carnivorous species

| Author | n.e | Mean.e | Sd.e | n.c | Mean.c | Sd.c |
| --- | --- | --- | --- | --- | --- | --- |
| Abdel Tawwab et al. [2] | 30 | 100 | 0 | 30 | 100 | 0 |
| Biswas et al. [3] | 15 | 95.6 | 3.8 | 15 | 84.4 | 15.4 |
| Biswas et al. [4] | 20 | 98.3 | 2.4 | 20 | 100 | 0 |
| Chen et al. [7] | 35 | 98.5 | 1.65 | 35 | 99.05 | 2.33 |
| Chen et al. [7] | 35 | 99.05 | 1.48 | 35 | 99.05 | 2.33 |
| Chen et al. [7] | 35 | 98.12 | 2.91 | 35 | 99.05 | 2.33 |
| Chen et al. [7] | 35 | 96.2 | 1.42 | 35 | 99.05 | 2.33 |
| Chen et al. [7] | 35 | 97.59 | 2.85 | 35 | 99.05 | 2.33 |
| Chen et al. [7] | 35 | 98.57 | 2.39 | 35 | 99.05 | 2.33 |
| Cui et al. [8] | 30 | 86.67 | 18.239 | 30 | 86.67 | 10.571 |
| Delamare-Deboutteville et al. [9] | 45 | 99.3 | 1.44 | 45 | 96.3 | 5.24 |
| Delamare-Deboutteville et al. [9] | 45 | 100 | 0 | 45 | 96.3 | 5.24 |
| Guo et al. [10] | 25 | 94 | 10 | 25 | 85 | 12.6 |
| Guo et al. [10] | 25 | 96 | 8.15 | 25 | 85 | 12.6 |
| Huang et al. [11] | 25 | 89.25 | 17 | 25 | 92 | 8.15 |
| Huang et al. [11] | 25 | 95 | 12.6 | 25 | 92 | 8.15 |
| Huang et al. [11] | 25 | 89 | 9.55 | 25 | 92 | 8.15 |
| Huangfu et al. [77] | 80 | 90.08 | 2.2 | 80 | 91.5 | 2.45 |
| Huangfu et al. [77] | 80 | 91.33 | 2.53 | 80 | 93.75 | 2.17 |
| Liu et al. [78] | 39 | 69.23 | 55.455 | 39 | 97.44 | 9.243 |
| Lu et al. [14] | 30 | 90.82 | 1.65 | 30 | 92.48 | 1.65 |
| Qi et al. [79] | 35 | 99 | 5.916 | 35 | 98.1 | 5.916 |
| Rhodes et al. [70] | 20 | 86.67 | 25.268 | 20 | 91.67 | 25.268 |
| Rhodes et al. [70] | 20 | 96.67 | 25.268 | 20 | 91.67 | 25.268 |
| Tibbettes et al. [18] | 35 | 100 | 0 | 35 | 100 | 0 |
| Tibbettes et al. [18] | 35 | 100 | 0 | 35 | 100 | 0 |
| Tibbettes et al. [18] | 35 | 100 | 0 | 35 | 100 | 0 |
| Wang et al. [80] | 35 | 94.28 | 4.95 | 35 | 98.09 | 1.65 |
| Wu et al. [73] | 100 | 87.67 | 8.8 | 100 | 87.67 | 29.1 |
| Wu et al. [73] | 100 | 89 | 5.8 | 100 | 87.67 | 29.1 |
| Xu et al. [20] | 30 | 100 | 0 | 30 | 100 | 0 |
| Xu et al. [20] | 30 | 100 | 0 | 30 | 100 | 0 |
| Yang et al. [21] | 12 | 100 | 0 | 12 | 94.4 | 9.6 |
| Yang et al. [71] | 12 | 100 | 0 | 12 | 100 | 0 |
| Yang et al. [71] | 12 | 100 | 0 | 12 | 100 | 0 |
| Yang et al. [71] | 12 | 100 | 0 | 12 | 100 | 0 |
| Yang et al. [71] | 12 | 100 | 0 | 12 | 100 | 0 |
| Yang et al. [72] | 12 | 91.6 | 8.3 | 12 | 94.4 | 9.6 |
| Yang et al. [72] | 12 | 94.4 | 4.8 | 12 | 94.4 | 9.6 |
| Yang et al. [72] | 12 | 97.2 | 4.8 | 12 | 94.4 | 9.6 |
| Yang et al. [72] | 12 | 100 | 0 | 12 | 94.4 | 9.6 |
| Yu et al. [22] | 20 | 97.5 | 46.197 | 20 | 97.5 | 46.197 |
| Zhang et al. [23] | 20 | 98.33 | 7.469 | 20 | 95 | 12.924 |
| Zhang et al. [23] | 20 | 98.33 | 7.469 | 20 | 95 | 12.924 |
| Zhang et al. [23] | 20 | 98.33 | 7.469 | 20 | 95 | 12.924 |
| Zhang et al. 2022b | 150 | 83.11 | 25.965 | 150 | 84.22 | 47.275 |
| Zhang et al. [24] | 10 | 76.67 | 10.531 | 10 | 73.33 | 10.531 |
| Zheng et al. [25] | 30 | 100 | 0 | 30 | 100 | 0 |
| Zheng et al. [25] | 30 | 100 | 0 | 30 | 100 | 0 |
| Zheng et al. [25] | 30 | 100 | 0 | 30 | 100 | 0 |
| Zhu et al. [26] | 30 | 100 | 0 | 30 | 100 | 0 |
| Zhu et al. [26] | 30 | 100 | 0 | 30 | 100 | 0 |
| Zhu et al. [26] | 30 | 100 | 0 | 30 | 100 | 0 |
| Zhu et al. [26] | 30 | 100 | 0 | 30 | 100 | 0 |
| Fu et al. [33] | 25 | 100 | 0 | 25 | 100 | 0 |
| Fu et al. [33] | 25 | 100 | 0 | 25 | 100 | 0 |
| Hardy et al. [27] | 35 | 98.9 | 5.324 | 35 | 96.2 | 5.324 |
| Hardy et al. [27] | 35 | 100 | 0 | 35 | 96.2 | 5.324 |
| Marchi et al. [32] | 45 | 96.3 | 1.3 | 45 | 97.8 | 2.2 |
| Marchi et al. [32] | 45 | 98.5 | 2.6 | 45 | 97.8 | 2.2 |
| Marchi et al. [32] | 45 | 97.8 | 0 | 45 | 97.8 | 2.2 |
| Ruiz et al. [30] | 30 | 93.3 | 2.7 | 30 | 95.8 | 3.2 |
| Ruiz et al. [30] | 30 | 95 | 1.9 | 30 | 95.8 | 3.2 |
| Zamani et al. [31] | 15 | 100 | 0 | 15 | 100 | 0 |
| Zamani et al. [31] | 15 | 100 | 0 | 15 | 100 | 0 |

**Supplementary Table 6**. Detailed parameters of 77 independent studies for the survival rate (SR) of omnivorous and herbivorous species

| Author | n.e | Mean.e | Sd.e | n.c | Mean.c | Sd.c |
| --- | --- | --- | --- | --- | --- | --- |
| Adeoye et al. [34] | 20 | 95 | 5 | 20 | 86.67 | 12.58 |
| Adeoye et al. [34] | 20 | 88.33 | 10.41 | 20 | 86.67 | 12.58 |
| Alloul et al. [35] | 100 | 88.3 | 4.9 | 100 | 83.8 | 5.1 |
| Alloul et al. [35] | 100 | 90.2 | 4.2 | 100 | 83.8 | 5.1 |
| Alloul et al. [35] | 100 | 84.5 | 7 | 100 | 83.8 | 5.1 |
| Alloul et al. [35] | 100 | 87.8 | 5.7 | 100 | 83.8 | 5.1 |
| Bertini et al. [36] | 45 | 99.3 | 1.3 | 45 | 98.5 | 2.6 |
| Bertini et al. [36] | 45 | 97 | 3.4 | 45 | 98.5 | 2.6 |
| Chama et al. [38] | 30 | 100 | 0 | 30 | 98.89 | 1.11 |
| Chama et al. [38] | 30 | 97.78 | 6.08 | 30 | 98.89 | 1.11 |
| Chama et al. [38] | 30 | 97.78 | 12.159 | 30 | 98.89 | 1.11 |
| Chama et al. [38] | 30 | 98.89 | 6.08 | 30 | 98.89 | 1.11 |
| Chama et al. [38] | 30 | 100 | 0 | 30 | 98.89 | 1.11 |
| Chen et al. [63] | 40 | 95.83 | 26.816 | 40 | 93.75 | 7.906 |
| Chen et al. [63] | 40 | 99.16 | 7.4 | 40 | 93.75 | 7.906 |
| Chen et al. [63] | 40 | 98.33 | 14.863 | 40 | 93.75 | 7.906 |
| Chen et al. [64] | 50 | 91.67 | 18.031 | 50 | 93.6 | 18.031 |
| Chen et al. [64] | 50 | 95.6 | 18.031 | 50 | 93.6 | 18.031 |
| Chen et al. [64] | 50 | 95.67 | 18.031 | 50 | 93.6 | 18.031 |
| Chen et al. [65] | 40 | 92.5 | 2.5 | 40 | 88.75 | 1.77 |
| Chen et al. [65] | 40 | 94.17 | 1.44 | 40 | 90 | 2.5 |
| Chen et al. [65] | 40 | 91.17 | 3.75 | 40 | 95.83 | 1.44 |
| Chen et al. [66] | 40 | 98.33 | 2.36 | 40 | 92.5 | 4.08 |
| Chen et al. [66] | 40 | 97.5 | 2.04 | 40 | 92.5 | 4.08 |
| Chen et al. [66] | 40 | 95.83 | 3.12 | 40 | 92.5 | 4.08 |
| Chen et al. [67] | 40 | 92.5 | 5.692 | 40 | 88.75 | 7.767 |
| Chen et al. [67] | 40 | 94.17 | 5.692 | 40 | 90 | 7.767 |
| Chen et al. [67] | 40 | 91.71 | 5.692 | 40 | 95.83 | 7.767 |
| Dai et al. [39] | 50 | 85.33 | 24.961 | 50 | 85.33 | 26.234 |
| Felix et al. [41] | 60 | 95.4 | 0.8 | 60 | 94.6 | 2.8 |
| Felix et al. [41] | 60 | 96.7 | 1.4 | 60 | 94.6 | 2.8 |
| Felix et al. [41] | 60 | 94.2 | 2.15 | 60 | 94.6 | 2.8 |
| Gisbert et al. [42] | 100 | 61 | 0.73 | 100 | 61.6 | 2.89 |
| Gisbert et al. [42] | 100 | 58.5 | 3 | 100 | 61.6 | 2.89 |
| Gisbert et al. [42] | 100 | 63.4 | 7.57 | 100 | 61.6 | 2.89 |
| Glengross et al. [43] | 30 | 97.8 | 3.725 | 30 | 96.7 | 3.725 |
| Glengross et al. [43] | 30 | 98.9 | 3.725 | 30 | 96.7 | 3.725 |
| Glengross et al. [43] | 30 | 98.9 | 3.725 | 30 | 96.7 | 3.725 |
| Glengross et al. [43] | 30 | 94.4 | 3.725 | 30 | 96.7 | 3.725 |
| Glengross et al. [43] | 30 | 94.4 | 3.725 | 30 | 96.7 | 3.725 |
| González-Félix et al. [44] | 10 | 93 | 15.147 | 10 | 95 | 15.811 |
| Hamidoghli et al. [45] | 50 | 73 | 10.607 | 50 | 72 | 10.607 |
| Hamidoghli et al. [45] | 50 | 77 | 10.607 | 50 | 72 | 10.607 |
| Jalal et al. [46] | 10 | 100 | 0 | 10 | 96.7 | 1.91 |
| Jiang et al. [47] | 40 | 83.25 | 2.97 | 40 | 87.67 | 5.03 |
| Jintasataporn et al. [48] | 25 | 89.6 | 2.19 | 25 | 85.6 | 6.07 |
| Jintasataporn et al. [48] | 25 | 89.6 | 6.07 | 25 | 85.6 | 6.07 |
| Jintasataporn et al. [48] | 25 | 89.6 | 3.58 | 25 | 85.6 | 6.07 |
| Kuo et al. [49] | 20 | 88.8 | 2.5 | 20 | 91.3 | 2.5 |
| Kuo et al. [49] | 20 | 85 | 0 | 20 | 91.3 | 2.5 |
| Li et al. [50] | 30 | 100 | 0 | 30 | 100 | 0 |
| Li et al. [50] | 30 | 100 | 0 | 30 | 100 | 0 |
| Li et al. [50] | 30 | 100 | 0 | 30 | 100 | 0 |
| Li et al. [50] | 30 | 100 | 0 | 30 | 100 | 0 |
| Li et al. [51] | 30 | 93.33 | 18.239 | 30 | 97.78 | 6.08 |
| Liao et al. [52] | 40 | 96.67 | 13.282 | 40 | 88.89 | 32.761 |
| Liao et al. [52] | 40 | 97.78 | 10.119 | 40 | 88.89 | 32.761 |
| Maulu et al. [53] | 30 | 97.78 | 2.629 | 30 | 98.89 | 2.629 |
| Maulu et al. [53] | 30 | 100 | 2.629 | 30 | 98.89 | 2.629 |
| Maulu et al. [53] | 30 | 100 | 2.629 | 30 | 98.89 | 2.629 |
| Maulu et al. [53] | 30 | 100 | 2.629 | 30 | 98.89 | 2.629 |
| Nederlof et al. [54] | 30 | 98 | 7.12 | 30 | 96 | 7.12 |
| Nederlof et al. [54] | 30 | 97 | 7.12 | 30 | 96 | 7.12 |
| Nederlof et al. [54] | 30 | 94 | 7.12 | 30 | 96 | 7.12 |
| Nederlof et al. [54] | 30 | 93 | 7.12 | 30 | 96 | 7.12 |
| Schneider et al. [56] | 40 | 100 | 2.087 | 40 | 100 | 2.087 |
| Shi et al. [57] | 40 | 98.33 | 1.44 | 40 | 98.33 | 2.89 |
| Shi et al. [57] | 40 | 99.17 | 1.44 | 40 | 98.33 | 2.89 |
| Wu et al. [81] | 60 | 95.89 | 1.084 | 60 | 95 | 0.155 |
| Yang et al. [75] | 50 | 91.33 | 33.022 | 50 | 87.33 | 4.738 |
| Yao et al. [76] | 40 | 85.67 | 2.31 | 40 | 87.67 | 5.03 |
| Yao et al. [76] | 40 | 85.67 | 2.31 | 40 | 87.67 | 5.03 |
| Yu et al. [61] | 30 | 98.33 | 6.244 | 30 | 98.89 | 3.834 |
| Yu et al. [61] | 30 | 96.67 | 6.682 | 30 | 98.89 | 3.834 |
| Yu et al. [61] | 30 | 96.67 | 9.421 | 30 | 98.89 | 3.834 |
| Yuan et al. [62] | 40 | 91.67 | 0.83 | 40 | 93.33 | 1.44 |
| Cai et al. [37] | 40 | 96.25 | 1.77 | 40 | 91.25 | 1.77 |

**Supplementary Table 7**. Detailed parameters of 88 independent studies for the hepatosomatic index (HSI) of aquaculture species

| Author | n.e | Mean.e | Sd.e | n.c | Mean.c | Sd.c |
| --- | --- | --- | --- | --- | --- | --- |
| Abdel Tawwab et al. [2] | 30 | 1.753 | 0.323 | 30 | 1.757 | 0.323 |
| Adeoye et al. [34] | 20 | 1.42 | 0.32 | 20 | 1.29 | 0.05 |
| Adeoye et al. [34] | 20 | 1.49 | 0.14 | 20 | 1.29 | 0.05 |
| Aas et al. [1] | 18 | 1.2 | 0.085 | 18 | 1.33 | 0.7 |
| Aas et al. [1] | 18 | 1.35 | 0.127 | 18 | 1.33 | 0.7 |
| Aas et al. [1] | 18 | 1.22 | 0.127 | 18 | 1.33 | 0.7 |
| Aas et al. [1] | 18 | 1.38 | 0.127 | 18 | 1.33 | 0.7 |
| Bai et al. [68] | 35 | 1.65 | 0.18 | 35 | 1.61 | 0.03 |
| Bai et al. [68] | 35 | 1.45 | 0.11 | 35 | 1.61 | 0.03 |
| Bertini et al. [36] | 45 | 0.97 | 0.15 | 45 | 1.07 | 0.24 |
| Bertini et al. [36] | 45 | 1.1 | 0.29 | 45 | 1.07 | 0.24 |
| Biswas et al. [4] | 20 | 1.9 | 0.4 | 20 | 1.7 | 0.1 |
| Biswas et al. [3] | 15 | 1.66 | 0.27 | 15 | 1.49 | 0.35 |
| Cai et al. [37] | 40 | 4.13 | 0.41 | 40 | 4.29 | 0.58 |
| Chen et al. [7] | 35 | 1.57 | 0.18 | 35 | 1.39 | 0.12 |
| Chen et al. [7] | 35 | 1.53 | 0.19 | 35 | 1.39 | 0.12 |
| Chen et al. [7] | 35 | 1.46 | 0.13 | 35 | 1.39 | 0.12 |
| Chen et al. [7] | 35 | 1.57 | 0.23 | 35 | 1.39 | 0.12 |
| Chen et al. [7] | 35 | 1.42 | 0.17 | 35 | 1.39 | 0.12 |
| Chen et al. [7] | 35 | 1.45 | 0.21 | 35 | 1.39 | 0.12 |
| Chen et al. [64] | 50 | 4.63 | 0.778 | 50 | 5.17 | 0.778 |
| Chen et al. [64] | 50 | 4.66 | 0.778 | 50 | 5.17 | 0.778 |
| Chen et al. [64] | 50 | 4.52 | 0.778 | 50 | 5.17 | 0.778 |
| Cui et al. [8] | 30 | 15.33 | 4.765 | 30 | 14.95 | 6.299 |
| Dai et al. [39] | 50 | 5.46 | 1.273 | 50 | 5.54 | 3.812 |
| Fu et al. [33] | 25 | 2.14 | 0.02 | 25 | 2.17 | 0.01 |
| Fu et al. [33] | 25 | 2.3 | 0.1 | 25 | 2.17 | 0.01 |
| Guo et al. [10] | 25 | 1.5 | 0.4 | 25 | 1.49 | 0.25 |
| Guo et al. [10] | 25 | 1.48 | 0.35 | 25 | 1.49 | 0.25 |
| Huang et al. [11] | 25 | 3.18 | 0.6 | 25 | 3.17 | 0.5 |
| Huang et al. [11] | 25 | 3.25 | 0.35 | 25 | 3.17 | 0.5 |
| Huang et al. [11] | 25 | 3.28 | 0.55 | 25 | 3.17 | 0.5 |
| Jintasataporn et al. [48] | 25 | 1.3 | 0.03 | 25 | 1.2 | 0.04 |
| Jintasataporn et al. [48] | 25 | 1.2 | 0.04 | 25 | 1.2 | 0.04 |
| Jintasataporn et al. [48] | 25 | 1.2 | 0.02 | 25 | 1.2 | 0.04 |
| Li et al. [50] | 30 | 1.2 | 0.274 | 30 | 1.17 | 0.493 |
| Li et al. [50] | 30 | 1.26 | 0.383 | 30 | 1.17 | 0.493 |
| Li et al. [50] | 30 | 1.11 | 0.219 | 30 | 1.17 | 0.493 |
| Li et al. [50] | 30 | 1.27 | 0.274 | 30 | 1.17 | 0.493 |
| Liao et al. [52] | 40 | 4.74 | 0.885 | 40 | 5.94 | 1.455 |
| Liao et al. [52] | 40 | 3.41 | 0.759 | 40 | 5.94 | 1.455 |
| Lu et al. [13] | 24 | 2.46 | 0.686 | 24 | 2.6 | 0.979 |
| Lu et al. [13] | 24 | 2.49 | 0.931 | 24 | 2.6 | 0.979 |
| Lund et al. [28] | 11 | 1.28 | 0.17 | 11 | 1.22 | 0.28 |
| Ma et al. [15] | 25 | 1.57 | 0.35 | 25 | 1.6 | 0.35 |
| Ma et al. [15] | 25 | 1.58 | 0.35 | 25 | 1.6 | 0.35 |
| Ma et al. [15] | 25 | 1.62 | 0.35 | 25 | 1.6 | 0.35 |
| Ma et al. [15] | 25 | 1.72 | 0.35 | 25 | 1.6 | 0.35 |
| Marchi et al. [32] | 45 | 1.35 | 0.24 | 45 | 1.22 | 0.22 |
| Marchi et al. [32] | 45 | 1.36 | 0.25 | 45 | 1.22 | 0.22 |
| Marchi et al. [32] | 45 | 1.31 | 0.26 | 45 | 1.22 | 0.22 |
| Papini et al. [29] | 19 | 1.45 | 0.392 | 19 | 1.33 | 0.619 |
| Papini et al. [29] | 19 | 1.42 | 0.789 | 19 | 1.33 | 0.619 |
| Ruiz et al. 2023 | 30 | 0.85 | 0.15 | 30 | 0.75 | 0.07 |
| Ruiz et al. 2023 | 30 | 0.74 | 0.13 | 30 | 0.75 | 0.07 |
| Tibbettes et al. [18] | 35 | 1.1 | 0.1 | 35 | 1 | 0.1 |
| Tibbettes et al. [18] | 35 | 1.2 | 0 | 35 | 1 | 0.1 |
| Tibbettes et al. [18] | 35 | 1.2 | 0 | 35 | 1 | 0.1 |
| Wang et al. [80] | 35 | 1.79 | 0.53 | 35 | 3.3 | 0.75 |
| Woolley et al. [19] | 13 | 2.1 | 0.361 | 13 | 2.3 | 0.361 |
| Woolley et al. [19] | 13 | 1.8 | 0.361 | 13 | 2.3 | 0.361 |
| Woolley et al. [19] | 13 | 1.8 | 0.361 | 13 | 2.3 | 0.361 |
| Wu et al. [73] | 100 | 3.06 | 0.9 | 100 | 2.96 | 1.2 |
| Wu et al. [73] | 100 | 3.04 | 0.5 | 100 | 2.96 | 1.2 |
| Xu et al. [20] | 30 | 2.47 | 0.657 | 30 | 2.55 | 0.657 |
| Xu et al. [20] | 30 | 2.56 | 0.329 | 30 | 2.55 | 0.657 |
| Yang et al. [71] | 12 | 2.48 | 0.09 | 12 | 2.59 | 0.18 |
| Yang et al. [71] | 12 | 2.49 | 0.09 | 12 | 2.59 | 0.18 |
| Yang et al. [71] | 12 | 2.35 | 0.13 | 12 | 2.59 | 0.18 |
| Yang et al. [71] | 12 | 2.28 | 0.1 | 12 | 2.59 | 0.18 |
| Yang et al. [72] | 12 | 3.49 | 0.39 | 12 | 3.29 | 0.38 |
| Yang et al. [72] | 12 | 2.88 | 0.39 | 12 | 3.29 | 0.38 |
| Yang et al. [72] | 12 | 2.85 | 0.22 | 12 | 3.29 | 0.38 |
| Yang et al. [72] | 12 | 3.59 | 0.41 | 12 | 3.29 | 0.38 |
| Yang et al. [75] | 50 | 3.66 | 0.071 | 50 | 3.57 | 0.495 |
| Zamani et al. [31] | 15 | 1.42 | 0.13 | 15 | 1.4 | 0.12 |
| Zamani et al. [31] | 15 | 1.42 | 0.14 | 15 | 1.4 | 0.12 |
| Zhang et al. [23] | 20 | 3.87 | 1.207 | 20 | 3.83 | 1.521 |
| Zhang et al. [23] | 20 | 3.86 | 1.834 | 20 | 3.83 | 1.521 |
| Zhang et al. [23] | 20 | 3.33 | 1.386 | 20 | 3.83 | 1.521 |
| Zhang et al. [82] | 150 | 2.6 | 1.102 | 150 | 2.24 | 2.082 |
| Zheng et al. [25] | 30 | 1.04 | 0.219 | 30 | 1.21 | 0.274 |
| Zheng et al. [25] | 30 | 1.25 | 0.329 | 30 | 1.21 | 0.274 |
| Zheng et al. [25] | 30 | 1.44 | 0.602 | 30 | 1.21 | 0.274 |
| Zhu et al. [26] | 30 | 7.03 | 1.863 | 30 | 7.35 | 0.164 |
| Zhu et al. [26] | 30 | 7.09 | 0.712 | 30 | 7.35 | 0.164 |
| Zhu et al. [26] | 30 | 6.95 | 0.219 | 30 | 7.35 | 0.164 |

**Supplementary Table 8**. Detailed parameters of 67 independent studies for the viscerosomatic index (VSI) of aquaculture species

| Author | n.e | Mean.e | Sd.e | n.c | Mean.c | Sd.c |
| --- | --- | --- | --- | --- | --- | --- |
| Abdel Tawwab et al. [2] | 30 | 0.215 | 0.307 | 30 | 0.219 | 0.285 |
| Adeoye et al. [34] | 20 | 10.18 | 0.28 | 20 | 10.51 | 1.08 |
| Adeoye et al. [34] | 20 | 10.35 | 0.35 | 20 | 10.51 | 1.08 |
| Aas et al. [1] | 18 | 6.91 | 0.679 | 18 | 7.37 | 0.552 |
| Aas et al. [1] | 18 | 6.93 | 0.212 | 18 | 7.37 | 0.552 |
| Aas et al. [1] | 18 | 7.44 | 0.509 | 18 | 7.37 | 0.552 |
| Aas et al. [1] | 18 | 7.52 | 0.382 | 18 | 7.37 | 0.552 |
| Bertini et al. [36] | 45 | 5.79 | 0.63 | 45 | 5.2 | 0.6 |
| Bertini et al. [36] | 45 | 5 | 1.19 | 45 | 5.2 | 0.6 |
| Biswas et al. [4] | 20 | 8.8 | 1.2 | 20 | 8.9 | 1.4 |
| Biswas et al. [3] | 15 | 5.7 | 0.41 | 15 | 5.03 | 0.5 |
| Cui et al. [8] | 30 | 19.24 | 4.71 | 30 | 19.04 | 7.449 |
| Dai et al. [39] | 50 | 18.56 | 6.081 | 50 | 17.94 | 10.96 |
| Fu et al. [33] | 25 | 6.41 | 0.19 | 25 | 6.4 | 0.06 |
| Fu et al. [33] | 25 | 6.56 | 0.27 | 25 | 6.4 | 0.06 |
| Guo et al. [10] | 25 | 7.06 | 1.15 | 25 | 7.03 | 0.9 |
| Guo et al. [10] | 25 | 7.67 | 1.5 | 25 | 7.03 | 0.9 |
| Huang et al. [11] | 25 | 9.91 | 0.65 | 25 | 9.6 | 1.1 |
| Huang et al. [11] | 25 | 10 | 1.65 | 25 | 9.6 | 1.1 |
| Huang et al. [11] | 25 | 10.01 | 0.2 | 25 | 9.6 | 1.1 |
| Li et al. [50] | 30 | 10.2 | 2.411 | 30 | 11.34 | 3.012 |
| Li et al. [50] | 30 | 9 | 1.807 | 30 | 11.34 | 3.012 |
| Li et al. [50] | 30 | 9.23 | 3.122 | 30 | 11.34 | 3.012 |
| Li et al. [50] | 30 | 8.91 | 1.369 | 30 | 11.34 | 3.012 |
| Lund et al. [28] | 11 | 12.48 | 2.91 | 11 | 12.32 | 1.29 |
| Ma et al. [15] | 25 | 7.98 | 0.8 | 25 | 8.5 | 0.8 |
| Ma et al. [15] | 25 | 8.27 | 0.8 | 25 | 8.5 | 0.8 |
| Ma et al. [15] | 25 | 7.9 | 0.8 | 25 | 8.5 | 0.8 |
| Ma et al. [15] | 25 | 8.16 | 0.8 | 25 | 8.5 | 0.8 |
| Marchi et al. [32] | 45 | 5.65 | 1.32 | 45 | 6.17 | 1.02 |
| Marchi et al. [32] | 45 | 5.98 | 0.77 | 45 | 6.17 | 1.02 |
| Marchi et al. [32] | 45 | 5.93 | 1.3 | 45 | 6.17 | 1.02 |
| Papini et al. [29] | 19 | 9.97 | 1.931 | 19 | 10.2 | 1.242 |
| Papini et al. [29] | 19 | 9.69 | 2.206 | 19 | 10.2 | 1.242 |
| Tibbettes et al. [18] | 35 | 9.4 | 0.7 | 35 | 9.5 | 0.5 |
| Tibbettes et al. [18] | 35 | 10.7 | 1.2 | 35 | 9.5 | 0.5 |
| Tibbettes et al. [18] | 35 | 10.7 | 0.8 | 35 | 9.5 | 0.5 |
| Wang et al. [80] | 35 | 7.18 | 0.6 | 35 | 9.5 | 0.89 |
| Woolley et al. [19] | 13 | 3.9 | 1.803 | 13 | 4 | 0.361 |
| Woolley et al. [19] | 13 | 4 | 1.442 | 13 | 4 | 0.361 |
| Woolley et al. [19] | 13 | 3.6 | 1.082 | 13 | 4 | 0.361 |
| Wu et al. [73] | 100 | 5.14 | 0.4 | 100 | 5.16 | 1.6 |
| Wu et al. [73] | 100 | 5.03 | 0.4 | 100 | 5.16 | 1.6 |
| Xu et al. [20] | 30 | 9.32 | 0.876 | 30 | 9.35 | 0.931 |
| Xu et al. [20] | 30 | 9.17 | 1.753 | 30 | 9.35 | 0.931 |
| Xue et al. [60] | 20 | 5.59 | 3.318 | 20 | 5.38 | 3.649 |
| Xue et al. [60] | 20 | 5.39 | 2.987 | 20 | 5.38 | 3.649 |
| Yang et al. [71] | 12 | 8.12 | 0.18 | 12 | 8.09 | 0.17 |
| Yang et al. [71] | 12 | 7.92 | 0.1 | 12 | 8.09 | 0.17 |
| Yang et al. [71] | 12 | 7.65 | 0.19 | 12 | 8.09 | 0.17 |
| Yang et al. [71] | 12 | 7.7 | 0.18 | 12 | 8.09 | 0.17 |
| Yang et al. [72] | 12 | 6.99 | 0.02 | 12 | 7.21 | 0.6 |
| Yang et al. [72] | 12 | 7.37 | 0.8 | 12 | 7.21 | 0.6 |
| Yang et al. [72] | 12 | 7.79 | 0.78 | 12 | 7.21 | 0.6 |
| Yang et al. [72] | 12 | 7.97 | 0.56 | 12 | 7.21 | 0.6 |
| Yang et al. [75] | 50 | 20.4 | 6.01 | 50 | 19.2 | 2.546 |
| Zhang et al. [23] | 20 | 9.22 | 2.504 | 20 | 9.09 | 2.057 |
| Zhang et al. [23] | 20 | 9.83 | 1.968 | 20 | 9.09 | 2.057 |
| Zhang et al. [23] | 20 | 9.14 | 1.163 | 20 | 9.09 | 2.057 |
| Zhang et al. [82] | 150 | 7.75 | 1.347 | 150 | 7.22 | 1.96 |
| Zheng et al. [25] | 30 | 5.22 | 0.493 | 30 | 5.38 | 0.602 |
| Zheng et al. [25] | 30 | 5.39 | 0.712 | 30 | 5.38 | 0.602 |
| Zheng et al. [25] | 30 | 5.45 | 0.548 | 30 | 5.38 | 0.602 |
| Zhu et al. [26] | 30 | 1.84 | 0.219 | 30 | 2.05 | 0.274 |
| Zhu et al. [26] | 30 | 1.89 | 0.383 | 30 | 2.05 | 0.274 |
| Zhu et al. [26] | 30 | 1.66 | 0.767 | 30 | 2.05 | 0.274 |

**Supplementary Table 9**. Detailed parameters of 49 independent studies for the  [alanine transaminase](https://www.google.com/search?sca_esv=6ddd0cbd40df43d1&rlz=1C1GCEU_enEG1119EG1119&q=alanine+transaminase&sa=X&sqi=2&ved=2ahUKEwjUmYroqsSQAxVzzgIHHQWUPf8QxccNegQIFhAB&mstk=AUtExfDu_wTJZm5iBcGTJnhnxg0M09UdVguyQwIIyQHtemMjZnWCCO0lBznkfvOQlJMOZEiduBS9cK6Mg7W0WFqP2P8Rpxi2YAG19aP-jDWydMzL5_15XuNbJYt2Qqeyf-qfRDafFAVx37m3p7YILpOA-1EIEbz7b9DXNgCgMNlFcUZABpT4NSwXKQHZSwPoDAxDp2RR&csui=3) activity (ALT) of aquaculture species

| Author | n.e | Mean.e | Sd.e | n.c | Mean.c | Sd.c |
| --- | --- | --- | --- | --- | --- | --- |
| Chen et al. [64] | 50 | 3.73 | 3.677 | 50 | 3.45 | 3.677 |
| Chen et al. [64] | 50 | 3.19 | 3.677 | 50 | 3.45 | 3.677 |
| Chen et al. [64] | 50 | 3.06 | 3.677 | 50 | 3.45 | 3.677 |
| Chen et al. [66] | 40 | 11.37 | 0.58 | 40 | 21.06 | 3.73 |
| Chen et al. [66] | 40 | 14.59 | 0.51 | 40 | 21.06 | 3.73 |
| Chen et al. [66] | 40 | 28.99 | 0.95 | 40 | 21.06 | 3.73 |
| Chen et al. [67] | 40 | 5.62 | 1.41 | 40 | 8.71 | 1.303 |
| Chen et al. [67] | 40 | 5.1 | 1.41 | 40 | 8.36 | 1.303 |
| Chen et al. [67] | 40 | 7.53 | 1.41 | 40 | 7.88 | 1.303 |
| Dai et al. [39] | 50 | 24.64 | 8.697 | 50 | 27.67 | 2.19 |
| Fu et al. [33] | 25 | 8.73 | 1.03 | 25 | 10.3 | 0.89 |
| Fu et al. [33] | 25 | 6 | 0.96 | 25 | 10.3 | 0.89 |
| Guo et al. [10] | 25 | 8.14 | 8 | 25 | 16.4 | 9.5 |
| Guo et al. [10] | 25 | 9.14 | 7.5 | 25 | 16.4 | 9.5 |
| Huang et al. [11] | 25 | 72.38 | 7.4 | 25 | 73.24 | 12.55 |
| Huang et al. [11] | 25 | 82.07 | 9.4 | 25 | 73.24 | 12.55 |
| Huang et al. [11] | 25 | 82.4 | 11.55 | 25 | 73.24 | 12.55 |
| Jiang et al. [47] | 40 | 11.54 | 1.07 | 40 | 38.03 | 0.76 |
| Lu et al. [13] | 24 | 2.89 | 1.037 | 24 | 3.8 | 1.02 |
| Lu et al. [13] | 24 | 7.04 | 4.786 | 24 | 3.8 | 1.02 |
| Ma et al. [15] | 25 | 42.9 | 10 | 25 | 62.6 | 10 |
| Ma et al. [15] | 25 | 45.5 | 10 | 25 | 62.6 | 10 |
| Ma et al. [15] | 25 | 44.5 | 10 | 25 | 62.6 | 10 |
| Ma et al. [15] | 25 | 44.4 | 10 | 25 | 62.6 | 10 |
| Marchi et al. [32] | 45 | 16.3 | 4.11 | 45 | 14.6 | 0.97 |
| Marchi et al. [32] | 45 | 31.3 | 6.36 | 45 | 14.6 | 0.97 |
| Marchi et al. [32] | 45 | 15.7 | 2.8 | 45 | 14.6 | 0.97 |
| Maulu et al. [53] | 30 | 20.56 | 10.681 | 30 | 27.39 | 10.681 |
| Maulu et al. [53] | 30 | 24.8 | 10.681 | 30 | 27.39 | 10.681 |
| Maulu et al. [53] | 30 | 27.6 | 10.681 | 30 | 27.39 | 10.681 |
| Maulu et al. [53] | 30 | 17.48 | 10.681 | 30 | 27.39 | 10.681 |
| Woolley et al. [19] | 13 | 20 | 6 | 13 | 22 | 11 |
| Woolley et al. [19] | 13 | 12 | 3 | 13 | 22 | 11 |
| Woolley et al. [19] | 13 | 28 | 14 | 13 | 22 | 11 |
| Wu et al. [81] | 60 | 15.06 | 5.112 | 60 | 14.47 | 3.408 |
| Wu et al. [59] | 30 | 4.23 | 0.95 | 30 | 10.77 | 10.28 |
| Wu et al. [59] | 30 | 4.67 | 1.3 | 30 | 3.53 | 0.65 |
| Wu et al. [59] | 30 | 4.1 | 3.38 | 30 | 2.23 | 0.55 |
| Yu et al. [61] | 30 | 10.8 | 5.751 | 30 | 20.09 | 12.489 |
| Yu et al. [61] | 30 | 19.06 | 10.899 | 30 | 20.09 | 12.489 |
| Yu et al. [61] | 30 | 19.23 | 12.707 | 30 | 20.09 | 12.489 |
| Zhang et al. [23] | 20 | 2.79 | 1.207 | 20 | 2.33 | 1.655 |
| Zhang et al. [23] | 20 | 2.81 | 2.012 | 20 | 2.33 | 1.655 |
| Zhang et al. [23] | 20 | 2.89 | 1.655 | 20 | 2.33 | 1.655 |
| Zhang et al. [24] | 10 | 22.59 | 4.996 | 10 | 24.46 | 8.918 |
| Zhu et al. [26] | 30 | 42.94 | 18.294 | 30 | 53.96 | 20.594 |
| Zhu et al. [26] | 30 | 44.14 | 24.099 | 30 | 53.96 | 20.594 |
| Zhu et al. [26] | 30 | 45.4 | 16.651 | 30 | 53.96 | 20.594 |

**Supplementary Table 10**. Detailed parameters of 49 independent studies for the aspartate aminotransferase (AST) activity of aquaculture species

| Author | n.e | Mean.e | Sd.e | n.c | Mean.c | Sd.c |
| --- | --- | --- | --- | --- | --- | --- |
| Chen et al. [64] | 50 | 3.79 | 3.041 | 50 | 3.49 | 3.041 |
| Chen et al. [64] | 50 | 3.12 | 3.041 | 50 | 3.49 | 3.041 |
| Chen et al. [64] | 50 | 3.5 | 3.041 | 50 | 3.49 | 3.041 |
| Chen et al. [66] | 40 | 15.06 | 0.27 | 40 | 21.31 | 3.24 |
| Chen et al. [66] | 40 | 18.97 | 2.44 | 40 | 21.31 | 3.24 |
| Chen et al. [66] | 40 | 31.43 | 2.21 | 40 | 21.31 | 3.24 |
| Chen et al. [67] | 40 | 7.38 | 3.624 | 40 | 8.32 | 2.479 |
| Chen et al. [67] | 40 | 6.58 | 3.624 | 40 | 7.03 | 2.479 |
| Chen et al. [67] | 40 | 6.68 | 3.624 | 40 | 10.77 | 2.479 |
| Dai et al. [39] | 50 | 14.63 | 1.909 | 50 | 15.03 | 1.909 |
| Fu et al. [33] | 25 | 276.63 | 7.21 | 25 | 259.57 | 8.5 |
| Fu et al. [33] | 25 | 232.5 | 6.01 | 25 | 259.57 | 8.5 |
| Guo et al. [10] | 25 | 6.47 | 3.75 | 25 | 8.24 | 3.9 |
| Guo et al. [10] | 25 | 8.53 | 3.15 | 25 | 8.24 | 3.9 |
| Huang et al. [11] | 25 | 16.86 | 14.65 | 25 | 14.44 | 5.5 |
| Huang et al. [11] | 25 | 17.5 | 10.35 | 25 | 14.44 | 5.5 |
| Huang et al. [11] | 25 | 21.46 | 3.2 | 25 | 14.44 | 5.5 |
| Jiang et al. [47] | 40 | 11.54 | 1.07 | 40 | 5.38 | 0.8 |
| Li et al. [50] | 30 | 273.43 | 67.589 | 30 | 300.83 | 34.999 |
| Li et al. [50] | 30 | 235.05 | 118.089 | 30 | 300.83 | 34.999 |
| Li et al. [50] | 30 | 247.73 | 121.978 | 30 | 300.83 | 34.999 |
| Li et al. [50] | 30 | 183.72 | 53.348 | 30 | 300.83 | 34.999 |
| Lu et al. [13] | 24 | 7.42 | 7.089 | 24 | 6.03 | 2.41 |
| Lu et al. [13] | 24 | 13.45 | 4.825 | 24 | 6.03 | 2.41 |
| Ma et al. [15] | 25 | 24.2 | 9.8 | 25 | 24.1 | 9.8 |
| Ma et al. [15] | 25 | 20.5 | 9.8 | 25 | 24.1 | 9.8 |
| Ma et al. [15] | 25 | 14.9 | 9.8 | 25 | 24.1 | 9.8 |
| Ma et al. [15] | 25 | 20.3 | 9.8 | 25 | 24.1 | 9.8 |
| Marchi et al. [32] | 45 | 58.8 | 15.3 | 45 | 59.6 | 8.49 |
| Marchi et al. [32] | 45 | 66.7 | 46.3 | 45 | 59.6 | 8.49 |
| Marchi et al. [32] | 45 | 62 | 12.8 | 45 | 59.6 | 8.49 |
| Woolley et al. [19] | 13 | 145 | 45 | 13 | 205 | 91 |
| Woolley et al. [19] | 13 | 86 | 27 | 13 | 205 | 91 |
| Woolley et al. [19] | 13 | 215 | 118 | 13 | 205 | 91 |
| Wu et al. [81] | 60 | 13.19 | 4.88 | 60 | 14.44 | 3.796 |
| Wu et al. [59] | 30 | 103.73 | 5.84 | 30 | 106.9 | 36.12 |
| Wu et al. [59] | 30 | 112.6 | 0.52 | 30 | 82.13 | 14.8 |
| Wu et al. [59] | 30 | 85.47 | 37.53 | 30 | 48.57 | 7.96 |
| Yu et al. [61] | 30 | 227.87 | 99.412 | 30 | 231.46 | 117.486 |
| Yu et al. [61] | 30 | 229.28 | 120.718 | 30 | 231.46 | 117.486 |
| Yu et al. [61] | 30 | 244.98 | 132.877 | 30 | 231.46 | 117.486 |
| Zhang et al. [23] | 20 | 36.34 | 13.416 | 20 | 34.66 | 10.018 |
| Zhang et al. [23] | 20 | 39.57 | 18.112 | 20 | 34.66 | 10.018 |
| Zhang et al. [23] | 20 | 46.56 | 18.34 | 20 | 34.66 | 10.018 |
| Zhang et al. [24] | 10 | 10.63 | 1.866 | 10 | 8.67 | 2.878 |
| Zhu et al. [26] | 30 | 17.02 | 4.984 | 30 | 22.76 | 7.12 |
| Zhu et al. [26] | 30 | 21.85 | 12.214 | 30 | 22.76 | 7.12 |
| Zhu et al. [26] | 30 | 26.03 | 5.97 | 30 | 22.76 | 7.12 |

**Supplementary Table 11**. Detailed parameters of 42 independent studies for the superoxide dismutase (SOD) activity of aquaculture species

| Author | n.e | Mean.e | Sd.e | n.c | Mean.c | Sd.c |
| --- | --- | --- | --- | --- | --- | --- |
| Chen et al. [64] | 50 | 0.43 | 0.495 | 50 | 0.48 | 0.495 |
| Chen et al. [64] | 50 | 0.43 | 0.495 | 50 | 0.48 | 0.495 |
| Chen et al. [64] | 50 | 0.5 | 0.495 | 50 | 0.48 | 0.495 |
| Chen et al. [67] | 40 | 126.51 | 48.946 | 40 | 71.2 | 2.448 |
| Chen et al. [67] | 40 | 130.09 | 48.946 | 40 | 112.22 | 2.448 |
| Chen et al. [67] | 40 | 148.23 | 48.946 | 40 | 121.03 | 2.448 |
| Fu et al. [33] | 25 | 22.19 | 1.01 | 25 | 23.32 | 0.39 |
| Fu et al. [33] | 25 | 21.73 | 1.38 | 25 | 23.32 | 0.39 |
| Gisbert et al. [42] | 100 | 71.77 | 5.83 | 100 | 52.77 | 5.35 |
| Gisbert et al. [42] | 100 | 89.57 | 3 | 100 | 52.77 | 5.35 |
| Gisbert et al. [42] | 100 | 90.95 | 1.77 | 100 | 52.77 | 5.35 |
| Guo et al. [10] | 25 | 18.6 | 6 | 25 | 13.6 | 5.2 |
| Guo et al. [10] | 25 | 17.7 | 11.2 | 25 | 13.6 | 5.2 |
| Huang et al. [11] | 25 | 8.63 | 0.35 | 25 | 9.9 | 1.9 |
| Huang et al. [11] | 25 | 9.59 | 2.3 | 25 | 9.9 | 1.9 |
| Huang et al. [11] | 25 | 8.66 | 2 | 25 | 9.9 | 1.9 |
| Jintasataporn et al. [48] | 25 | 26.37 | 3.78 | 25 | 26.13 | 3.13 |
| Jintasataporn et al. [48] | 25 | 23.92 | 3 | 25 | 26.13 | 3.13 |
| Li et al. [50] | 30 | 128.35 | 24.319 | 30 | 128.62 | 15.008 |
| Li et al. [50] | 30 | 126.78 | 15.281 | 30 | 128.62 | 15.008 |
| Li et al. [50] | 30 | 133.05 | 15.939 | 30 | 128.62 | 15.008 |
| Li et al. [50] | 30 | 124.87 | 21.197 | 30 | 128.62 | 15.008 |
| Lu et al. [13] | 24 | 551.17 | 158.477 | 24 | 656.92 | 207.232 |
| Lu et al. [13] | 24 | 449.14 | 268.185 | 24 | 656.92 | 207.232 |
| Lu et al. [14] | 30 | 38.66 | 1.66 | 30 | 38.68 | 2.23 |
| Rizwan et al. [17] | 10 | 278.46 | 33.789 | 10 | 271.12 | 40.123 |
| Xu et al. [20] | 30 | 142.04 | 30.234 | 30 | 142.18 | 38.505 |
| Xu et al. [20] | 30 | 134.54 | 25.524 | 30 | 142.18 | 38.505 |
| Yang et al. [71] | 12 | 145.1 | 5.1 | 12 | 146.9 | 13.6 |
| Yang et al. [71] | 12 | 164 | 1.7 | 12 | 146.9 | 13.6 |
| Yang et al. [71] | 12 | 165 | 7.1 | 12 | 146.9 | 13.6 |
| Yang et al. [71] | 12 | 144.1 | 7.1 | 12 | 146.9 | 13.6 |
| Yang et al. [75] | 50 | 46.06 | 30.448 | 50 | 34.56 | 27.867 |
| Yu et al. [61] | 30 | 23.06 | 8.709 | 30 | 23.97 | 7.559 |
| Yu et al. [61] | 30 | 22.93 | 7.066 | 30 | 23.97 | 7.559 |
| Yu et al. [61] | 30 | 22.27 | 4.327 | 30 | 23.97 | 7.559 |
| Yuan et al. [62] | 40 | 140.72 | 12.792 | 40 | 106.71 | 8.113 |
| Zhang et al. [24] | 10 | 197.17 | 39.462 | 10 | 227.54 | 49.986 |
| Zhu et al. [26] | 30 | 36.28 | 9.421 | 30 | 37.48 | 31.439 |
| Zhu et al. [26] | 30 | 36.88 | 9.421 | 30 | 37.48 | 31.439 |
| Zhu et al. [26] | 30 | 50.47 | 48.693 | 30 | 37.48 | 31.439 |

**Supplementary Table 12**. Detailed parameters of 25 independent studies for the catalase (CAT) activity of aquaculture species

| Author | n.e | Mean.e | Sd.e | n.c | Mean.c | Sd.c |
| --- | --- | --- | --- | --- | --- | --- |
| Chen et al. [64] | 50 | 0.32 | 0.283 | 50 | 0.33 | 0.283 |
| Chen et al. [64] | 50 | 0.37 | 0.283 | 50 | 0.33 | 0.283 |
| Chen et al. [64] | 50 | 0.36 | 0.283 | 50 | 0.33 | 0.283 |
| Chen et al. [67] | 40 | 47.34 | 16.57 | 40 | 35.8 | 24.369 |
| Chen et al. [67] | 40 | 60.28 | 16.57 | 40 | 51.95 | 24.369 |
| Chen et al. [67] | 40 | 50.36 | 16.57 | 40 | 49.29 | 24.369 |
| Fu et al. [33] | 25 | 2.21 | 0.18 | 25 | 2.73 | 0.71 |
| Fu et al. [33] | 25 | 2.4 | 0.2 | 25 | 2.73 | 0.71 |
| Gisbert et al. [42] | 100 | 22.87 | 0.15 | 100 | 14.55 | 0.11 |
| Gisbert et al. [42] | 100 | 30.22 | 0.09 | 100 | 14.55 | 0.11 |
| Gisbert et al. [42] | 100 | 31.33 | 0.15 | 100 | 14.55 | 0.11 |
| Li et al. [50] | 30 | 13.12 | 6.627 | 30 | 5.62 | 4.875 |
| Li et al. [50] | 30 | 15.4 | 7.723 | 30 | 5.62 | 4.875 |
| Li et al. [50] | 30 | 15.58 | 8.764 | 30 | 5.62 | 4.875 |
| Li et al. [50] | 30 | 15.16 | 6.901 | 30 | 5.62 | 4.875 |
| Lu et al. [13] | 24 | 12.04 | 3.416 | 24 | 7.99 | 2.466 |
| Lu et al. [13] | 24 | 14.13 | 1.387 | 24 | 7.99 | 2.466 |
| Lu et al. [14] | 30 | 3.45 | 0.64 | 30 | 3.4 | 0.46 |
| Yang et al. [75] | 50 | 13.79 | 7.637 | 50 | 9.73 | 4.575 |
| Yuan et al. [62] | 40 | 65.16 | 9.838 | 40 | 40 | 3.065 |
| Zhang et al. [24] | 10 | 2.02 | 0.484 | 10 | 2.03 | 0.613 |
| Zhu et al. [26] | 30 | 70.89 | 22.73 | 30 | 79.83 | 6.518 |
| Zhu et al. [26] | 30 | 59.29 | 3.834 | 30 | 79.83 | 6.518 |

**References**

[1] T.S. Aas, B. Grisdale-Helland, B.F. Terjesen, S.J. Helland, Improved growth and nutrient utilisation in Atlantic salmon (*Salmo salar*) fed diets containing a bacterial protein meal, Aquaculture 259 (2006) 365–376. https://doi.org/10.1016/j.aquaculture.2006.05.032.

[2] M. Abdel-Tawwab, R.H. Khalil, T.A.M. Abo Selema, M. Abdelsalam, T.M.N. Abdelhakim, E.A. Sabry, H.A. Abd El-Ghaffar, A.H. Saad, Evaluating the inclusion of *Clostridium autoethanogenum* protein instead of fishmeal protein in diets for European seabass (*Dicentrarchus labrax*): Growth performance, digestive enzymes, health status, and tissues investigations, Anim. Feed Sci. Technol. 324 (2025) 116318. https://doi.org/10.1016/j.anifeedsci.2025.116318.

[3] A. Biswas, F. Takakuwa, S. Yamada, A. Matsuda, R.M. Saville, A. Leblanc, J.A. Silverman, N. Sato, H. Tanaka, Methanotroph ( *Methylococcus capsulatus*, Bath ) bacteria meal as an alternative protein source for Japanese yellowtail, *Seriola quinqueradiata*, Aquaculture 529 (2020) 735700. https://doi.org/10.1016/j.aquaculture.2020.735700.

[4] A. Biswas, F. Takakuwa, S. Yamada, A. Furukawa, M. Shiratori, T. Koshiishi, H. Tomokane, H. Tanaka, Partial replacement of fishmeal by a protein-rich product from methanotrophic bacteria in juvenile red sea bream (*Pagrus major*), Aquac. Nutr. 27 (2021) 2726–2738. https://doi.org/10.1111/anu.13398.

[5] M. Carvalho, S. Torrecillas, D. Montero, A. Sanmartín, R. Fontanillas, A. Farías, K. Moutou, J.H. Velásquez, M. Izquierdo, Insect and single-cell protein meals as replacers of fish meal in low fish meal and fish oil diets for gilthead sea bream (*Sparus aurata*) juveniles, Aquaculture 566 (2023) 739215. https://doi.org/10.1016/j.aquaculture.2022.739215.

[6] M. Carvalho, R. Ginés, I. Martín, M.J. Zamorano, F. Acosta, R. Fontanillas, S. Torrecillas, D. Montero, Genetic selection for high growth improves the efficiency of gilthead sea bream (*Sparus aurata*) in using novel diets with insect meal, single-cell protein and a DHA rich-microalgal oil, Aquaculture 578 (2024) 740034. https://doi.org/10.1016/j.aquaculture.2023.740034.

[7] Y. Chen, G. Sagada, B. Xu, W. Chao, F. Zou, W.K. Ng, Y. Sun, L. Wang, Z. Zhong, Q. Shao, Partial replacement of fishmeal with *Clostridium autoethanogenum* single-cell protein in the diet for juvenile black sea bream (*Acanthopagrus schlegelii*), Aquac. Res. 51 (2020) 1000–1011. https://doi.org/10.1111/are.14446.

[8] X. Cui, Q. Ma, M. Duan, H. Xu, M. Liang, Y. Wei, Effects of fishmeal replacement by *Clostridium autoethanogenum* protein on the growth, digestibility, serum free amino acid and gene expression related to protein metabolism of obscure pufferfish ( *Takifugu obscurus* ), Anim. Feed Sci. Technol. 292 (2022) 115445. https://doi.org/10.1016/j.anifeedsci.2022.115445.

[9] J. Delamare-Deboutteville, D.J. Batstone, M. Kawasaki, S. Stegman, M. Salini, S. Tabrett, R. Smullen, A.C. Barnes, T. Hülsen, Mixed culture purple phototrophic bacteria is an effective fishmeal replacement in aquaculture, Water Res. X 4 (2019) 100031. https://doi.org/10.1016/j.wroa.2019.100031.

[10] B. Guo, X. He, C. Ge, M. Xue, J. Wang, M. Longshaw, J. Wang, X. Liang, A Natural Gas Fermentation Bacterial Meal ( FeedKind ® ) as a Functional Alternative Ingredient for Fishmeal in Diet of Largemouth Bass, *Micropterus salmoides*, Antioxidants 11 (2022) 1479. https://doi.org/https://doi.org/10.3390/ antiox11081479.

[11] H. Huang, X. Li, K. Cao, X. Leng, Effects of Replacing Fishmeal with the Mixture of Cottonseed Protein Concentrate and *Clostridium autoethanogenum*, Animals 13 (2023) 817. https://doi.org/https:// doi.org/10.3390/ani13050817.

[12] A. Kiessling, S. Askbrandt, Nutritive value of two bacterial strains of single-cell protein for rainbow trout (*Oncorhynchus mykiss*), Aquaculture 109 (1993) 119–130. https://doi.org/10.1016/0044-8486(93)90209-H.

[13] Q. Lu, L. Xi, Y. Liu, Y. Gong, J. Su, D. Han, Y. Yang, J. Jin, H. Liu, X. Zhu, S. Xie, Effects of Dietary Inclusion of *Clostridium autoethanogenum* Protein on the Growth Performance and Liver Health of Largemouth Bass (*Micropterus salmoides*), Front. Mar. Sci. 8 (2021) 1–12. https://doi.org/10.3389/fmars.2021.764964.

[14] W. Lu, H. Yu, Y. Liang, S. Zhai, Evaluation of Methanotroph (*Methylococcus capsulatus*, Bath) Bacteria Protein as an Alternative to Fish Meal in the Diet of Juvenile American Eel (*Anguilla rostrata*), Animals 13 (2023) 681. https://doi.org/10.3390/ani13040681.

[15] S. Ma, X. Liang, P. Chen, J. Wang, X. Gu, Y. Qin, C. Blecker, M. Xue, A new single-cell protein from *Clostridium autoethanogenum* as a functional protein for largemouth bass ( *Micropterus salmoides* ), Anim. Nutr. 10 (2022) 99–100. https://doi.org/10.1016/j.aninu.2022.04.005.

[16] L.W. Pilmer, L.D. Woolley, A.J. Lymbery, M. Salini, G.J. Partridge, Using dietary additives to improve palatability of diets containing single-cell protein from methanotrophic bacteria in yellowtail kingfish (*Seriola lalandi*) diets, Aquac. Res. 53 (2022) 5006–5017. https://doi.org/10.1111/are.15986.

[17] M. Rizwan, L. Li, G. Yang, Z. Zhang, C. Wang, Evaluation of flesh quality and antioxidant responses in grow-out largemouth bass ( *Micropterus salmoides* ) with novel protein substitutes, Aquac. Fish. (2025) 1–13. https://doi.org/https://doi.org/10.1016/j.aaf.2025.06.002.

[18] S.M. Tibbetts, M.J. Piercey, S.J.J. Patelakis, B. Stratton, Single-cell protein (SCP) meals from *Methylovorus menthalis* as feed ingredients for freshwater Atlantic salmon (*Salmo salar* L.): Digestibility, growth performance, nutrient utilization, and fish health, Aquaculture 598 (2025) 741874. https://doi.org/10.1016/j.aquaculture.2024.741874.

[19] L. Woolley, R. Chaklader, L. Pilmer, F. Stephens, C. Wingate, M. Salini, G. Partridge, Gas to protein : Microbial single cell protein is an alternative to fishmeal in aquaculture, Sci. Total Environ. 859 (2023) 160141. https://doi.org/10.1016/j.scitotenv.2022.160141.

[20] B. Xu, Y. Liu, K. Chen, L. Wang, G. Sagada, A.F. Tegomo, Y. Yang, Y. Sun, L. Zheng, S. Ullah, Q. Shao, Evaluation of Methanotroph (*Methylococcus capsulatus*, Bath) Bacteria Meal (FeedKind®) as an Alternative Protein Source for Juvenile Black Sea Bream, *Acanthopagrus schlegelii*, Front. Mar. Sci. 8 (2021) 778301. https://doi.org/10.3389/fmars.2021.778301.

[21] P. Yang, W. Yao, Y. Wang, M. Li, X. Li, X. Leng, Dietary effects of fish meal substitution with *Clostridium autoethanogenum* on flesh quality and metabolomics of largemouth bass (*Micropterus salmoides*), Aquac. Reports 23 (2022) 101012. https://doi.org/10.1016/j.aqrep.2022.101012.

[22] M. han Yu, X. shan Li, J. Wang, M. Longshaw, K. Song, L. Wang, C. xiao Zhang, K. le Lu, Substituting fish meal with a bacteria protein (*Methylococcus capsulatus*, Bath) grown on natural gas: Effects on growth non-specific immunity and gut health of spotted seabass (*Lateolabrax maculatus*), Anim. Feed Sci. Technol. 296 (2023) 115556. https://doi.org/10.1016/j.anifeedsci.2022.115556.

[23] Q. Zhang, H. Liang, M. Longshaw, J. Wang, X. Ge, J. Zhu, S. Li, M. Ren, Effects of replacing fishmeal with methanotroph (*Methylococcus capsulatus*, Bath) bacteria meal (FeedKind®) on growth and intestinal health status of juvenile largemouth bass (*Micropterus salmoides*), Fish Shellfish Immunol. 122 (2022) 298–305. https://doi.org/10.1016/j.fsi.2022.02.008.

[24] Y. Zhang, Y. Gu, Y. Tian, W. Zhan, Y. Deng, S. Xie, H. Peng, P. Sun, T. Zhu, M. Jin, Q. Zhou, Evaluation of *Clostridium autoethanogenum* protein as a fish meal substitute in diets for juvenile mud crab (*Scylla paramamosain*), Aquac. Reports 45 (2025) 103104. https://doi.org/10.1016/j.aqrep.2025.103104.

[25] J. Zheng, W. Zhang, Z. Dan, Y. Zhuang, Y. Liu, K. Mai, Q. Ai, Replacement of dietary fish meal with *Clostridium autoethanogenum* meal on growth performance, intestinal amino acids transporters, protein metabolism and hepatic lipid metabolism of juvenile turbot (*Scophthalmus maximus* L.), Front. Physiol. 13 (2022) 1–19. https://doi.org/10.3389/fphys.2022.981750.

[26] S. Zhu, W. Gao, Z. Wen, S. Chi, Y. Shi, W. Hu, B. Tan, Partial substitution of fish meal by *Clostridium autoethanogenum* protein in the diets of juvenile largemouth bass ( *Micropterus salmoides* ), Aquac. Reports 22 (2022) 100938. https://doi.org/10.1016/j.aqrep.2021.100938.

[27] R.W. Hardy, B. Patro, C. Pujol-Baxley, C.J. Marx, L. Feinberg, Partial replacement of soybean meal with *Methylobacterium extorquens* single-cell protein in feeds for rainbow trout (*Oncorhynchus mykiss* Walbaum), Aquac. Res. 49 (2018) 2218–2224. https://doi.org/10.1111/are.13678.

[28] I. Lund, P. Gómez-Requeni, J. Holm, N.W. Thorringer, Replacement of fish meal with a methanogen bacterial single cell protein: Effects on nutrient utilization and performance in juvenile rainbow trout (*Oncorhynchus mykiss*), Aquac. Reports 44 (2025). https://doi.org/10.1016/j.aqrep.2025.103042.

[29] G. Papini, A.G. Opinion, J. Desmidt, A. Alloul, P. Vermeir, G. De Boeck, S.E. Vlaeminck, Successful high replacement of fishmeal in rainbow trout feed by consortia of aerobic heterotrophic bacteria results in equal growth, health, and muscle composition, Aquac. Int. 33 (2025) 1–22. https://doi.org/10.1007/s10499-025-01955-4.

[30] A. Ruiz, I. Sanahuja, N.W. Thorringer, J. Lynegaard, E. Ntokou, D. Furones, E. Gisbert, Single cell protein from methanotrophic bacteria as an alternative healthy and functional protein source in aquafeeds, a holistic approach in rainbow trout (*Oncorhynchus mykiss*) juveniles, Aquaculture 576 (2023) 739861. https://doi.org/10.1016/j.aquaculture.2023.739861.

[31] A. Zamani, M. Khajavi, M.H. Nazarpak, E. Gisbert, Evaluation of a bacterial single-cell protein in compound diets for rainbow trout (*Oncorhynchus mykiss*) fry as an alternative protein source, Animals 10 (2020) 1–18. https://doi.org/10.3390/ani10091676.

[32] A. Marchi, A. Bonaldo, D. Scicchitano, M. Candela, A. De Marco, S. Falciglia, M. Mazzoni, G. Lattanzio, P. Clavenzani, F. Dondi, P.P. Gatta, L. Parma, Feeding gilthead sea bream with increasing dietary bacterial single cell protein level: Implication on growth, plasma biochemistry, gut histology, and gut microbiota, Aquaculture 565 (2023) 739132. https://doi.org/10.1016/j.aquaculture.2022.739132.

[33] Y. Fu, J. Wei, H. Lin, J. Zhang, Y. Zhang, J. Yu, J. Li, M. Xie, X. Shao, J. Ye, H. Mi, C. Wu, L. Zhang, Effects of fish meal replacement by *Clostridium autoethanogenum* protein on growth performance, serum biochemistry, antioxidant capacity, immune responses and muscle quality in black carp (*Mylopharyngodon piceus*), Aquac. Reports 43 (2025) 102892. https://doi.org/10.1016/j.aqrep.2025.102892.

[34] A.A. Adeoye, Y. Akegbejo-Samsons, F.J. Fawole, P.O. Olatunji, N. Muller, A.H.L. Wan, S.J. Davies, From waste to feed: Dietary utilisation of bacterial protein from fermentation of agricultural wastes in African catfish (*Clarias gariepinus*) production and health, Aquaculture 531 (2021) 735850. https://doi.org/10.1016/j.aquaculture.2020.735850.

[35] A. Alloul, M. Wille, P. Lucenti, P. Bossier, G. Van Stappen, S.E. Vlaeminck, Purple bacteria as added-value protein ingredient in shrimp feed: *Penaeus vannamei* growth performance, and tolerance against *Vibrio* and ammonia stress, Aquaculture 530 (2021) 735788. https://doi.org/10.1016/j.aquaculture.2020.735788.

[36] A. Bertini, S. Natale, E. Gisbert, K.B. Andrée, D. Concu, F. Dondi, A. De Cesare, V. Indio, P.P. Gatta, A. Bonaldo, L. Parma, Exploring the application of *Corynebacterium glutamicum* single cell protein in the diet of flathead grey mullet (*Mugil cephalus*): effects on growth performance, digestive enzymes activity and gut microbiota, Front. Mar. Sci. 10 (2023) 1–17. https://doi.org/10.3389/fmars.2023.1172505.

[37] Y. Cai, H. Huang, W. Yao, H. Yang, M. Xue, X. Li, X. Leng, Effects of fish meal replacement by three protein sources on physical pellet quality and growth performance of Pacific white shrimp (*Litopenaeus vannamei*), Aquac. Reports 25 (2022) 101210. https://doi.org/10.1016/j.aqrep.2022.101210.

[38] M.K.H. Chama, H. Liang, D. Huang, X. Ge, M. Ren, L. Zhang, L. Wu, J. Ke, Methanotroph (*Methylococcus capsulatus*, Bath) as an alternative protein source for genetically improved farmed tilapia (GIFT: *Oreochromis niloticus*) and its effect on antioxidants and immune response, Aquac. Reports 21 (2021) 100872. https://doi.org/10.1016/j.aqrep.2021.100872.

[39] J. Dai, T. Chen, X. Guo, Z. Dai, Z. He, Y. Hu, Evaluation of fish meal replacement by *Clostridium autoethanogenum* protein in diets for juvenile red swamp crayfish (*Procambarus clarkii*), Aquaculture 570 (2023) 739379. https://doi.org/10.1016/j.aquaculture.2023.739379.

[40] Z. Fan, C. Li, D. Wu, J. Li, L. Wang, D. Cao, L. Miao, S. Xie, Evaluation of four novel protein sources as alternatives to soybean meal for two specifications of cage-farmed grass carp (*Ctenopharyngodon idellus*) deeds: Effect on growth performance, flesh quality, and expressions of muscle-related genes, Front. Mar. Sci. 9 (2022) 1–15. https://doi.org/10.3389/fmars.2022.935651.

[41] N. Felix, K. Manikandan, A. Uma, S.J. Kaushik, Evaluation of single cell protein on the growth performance, digestibility and immune gene expression of Pacific white shrimp, *Penaeus vannamei*, Anim. Feed Sci. Technol. 296 (2023) 115549. https://doi.org/10.1016/j.anifeedsci.2022.115549.

[42] E. Gisbert, A. Ruiz, S. Torrecillas, Y. Cruz-Quintana, E. Bertomeu, C. Saromines, F. Melenchón, E. Ntokou, I. Pascual, S. Reinoso, S. Sarih, M.M. Solovyev, M.D. Furones, N.W. Thorringer, Methanotrophic bacterial meal as an alternative feed ingredient for whiteleg shrimp (*Litopenaeus vannamei*) diets, Aquac. Reports 45 (2025) 103094. https://doi.org/10.1016/j.aqrep.2025.103094.

[43] B. Glencross, S. Irvin, S. Arnold, D. Blyth, N. Bourne, N. Preston, Effective use of microbial biomass products to facilitate the complete replacement of fishery resources in diets for the black tiger shrimp, *Penaeus monodon*, Aquaculture 431 (2014) 12–19. https://doi.org/10.1016/j.aquaculture.2014.02.033.

[44] M.L. González-félix, R.D. Félix-berumen, M. Perez-velazquez, Use of bacterial-based single-cell protein MRD-Pro ® in diets for Nile tilapia ( *Oreochromis niloticus* ) fry, Arch. Biol. Sci. 76 (2024) 191–203. https://doi.org/https://doi.org/10.2298/ABS240403013G.

[45] A. Hamidoghli, H. Yun, S. Won, S.K. Kim, N.W. Farris, S.C. Bai, Evaluation of a single-cell protein as a dietary fish meal substitute for whiteleg shrimp *Litopenaeus vannamei*, Fish. Sci. 85 (2019) 147–155. https://doi.org/10.1007/s12562-018-1275-5.

[46] A.J.K. Chowdhury, N.H. Zakaria, Z.A.Z. Abidin, M.M. Rahman, Phototrophic purple bacteria as feed supplement on the growth, feed utilization and body compositions of Malaysian Mahseer, *Tor tambroides* juveniles, Sains Malaysiana 45 (2016) 135–140.

[47] X. Jiang, W. Yao, H. Yang, S. Tan, X. Leng, X. Li, Dietary effects of *Clostridium autoethanogenum* protein substituting fish meal on growth, intestinal histology and immunity of Pacific white shrimp (*Litopenaeus vannamei*) based on transcriptome analysis, Fish Shellfish Immunol. 119 (2021) 635–644. https://doi.org/10.1016/j.fsi.2021.10.005.

[48] O. Jintasataporn, S. Chumkam, S. Triwutanon, A. LeBlanc, J. Sawanboonchun, Effects of a Single Cell Protein (*Methylococcus capsulatus*, Bath) in Pacific White Shrimp (*Penaeus vannamei*) Diet on Growth Performance, Survival Rate and Resistance to Vibrio parahaemolyticus, the Causative Agent of Acute Hepatopancreatic Necrosis Diseas, Front. Mar. Sci. 8 (2021) 1–10. https://doi.org/10.3389/fmars.2021.764042.

[49] Y.C. Kuo, T.H. Ho, A. Bharadwaj, H.T.Q. Tran, Y.T. Chu, S.H. Wang, T.Y. Chen, F.H. Nan, P.T. Lee, Feasibility assessment of replacing fishmeal with *Clostridium autoethanogenum* protein in commercial whiteleg shrimp diets: Impacts on growth, muscle characteristics, and health, Anim. Feed Sci. Technol. 309 (2024) 115916. https://doi.org/10.1016/j.anifeedsci.2024.115916.

[50] M. Li, H. Liang, J. Xie, W. Chao, F. Zou, X. Ge, M. Ren, Diet supplemented with a novel *Clostridium autoethanogenum* protein have a positive effect on the growth performance, antioxidant status and immunity in juvenile Jian carp (*Cyprinus carpio* var. Jian), Aquac. Reports 19 (2021) 100572. https://doi.org/10.1016/j.aqrep.2020.100572.

[51] X. Li, Y. Chen, C. Zheng, S. Chi, S. Zhang, B. Tan, S. Xie, Evaluation of Six Novel Protein Sources on Apparent Digestibility in Pacific White Shrimp, *Litopenaeus vannamei*, Aquac. Nutr. 2022 (2022) 1–11. https://doi.org/10.1155/2022/8225273.

[52] Z. Liao, Y. Gong, W. Zhao, X. He, D. Wei, J. Niu, Comparison effect of *Rhodobacter sphaeroides* protein replace fishmeal on growth performance, intestinal morphology, hepatic antioxidant capacity and immune gene expression of *Litopenaeus vannamei* under low salt stress, Aquaculture 547 (2022) 737488. https://doi.org/10.1016/j.aquaculture.2021.737488.

[53] S. Maulu, H. Liang, X. Ge, H. Yu, D. Huang, J. Ke, M. Ren, H. Mi, Effect of dietary *Clostridium autoethanogenum* protein on growth, body composition, plasma parameters and hepatic genes expression related to growth and AMPK/TOR/PI3K signaling pathway of the genetically improved farmed tilapia (GIFT: *Oreochromis niloticus*, Anim. Feed Sci. Technol. 276 (2021) 114914. https://doi.org/10.1016/j.anifeedsci.2021.114914.

[54] M.A.J. Nederlof, S.J. Kaushik, J.W. Schrama, Effect of different types of bacterial single cell protein on feed intake, digestibility, growth and body composition of Pacific white shrimp (*Penaeus vannamei*), Aquac. Reports 33 (2023) 101830. https://doi.org/10.1016/j.aqrep.2023.101830.

[55] M.J. Salini, W. Wang, T.S. Mock, M.A.B. Siddik, M.K. Jago, K.L.F. Bews, D.S. Francis, Expanding the Ingredient Basket in Aquaculture: Growth Performance and Feed Utilization of Australian Hybrid Abalone (*Haliotis laevigata × H. rubra*) Fed Methanotrophic Single Cell Protein, Aquac. Nutr. 2025 (2025) 1–16. https://doi.org/10.1155/anu/7291857.

[56] O. Schneider, A.K. Amirkolaie, J. Vera-Cartas, E.H. Eding, J.W. Schrama, J.A.J. Verreth, Digestibility, faeces recovery, and related carbon, nitrogen and phosphorus balances of five feed ingredients evaluated as fishmeal alternatives in Nile tilapia, *Oreochromis niloticus* L., Aquac. Res. 35 (2004) 1370–1379. https://doi.org/10.1111/j.1365-2109.2004.01179.x.

[57] L. Shi, X. Yan, Z. Yan, H. Wang, J. Chen, S. Zhang, Combined microbiomic and transcriptomic analysis revealed that dietary *Clostridium autoethanogenum* protein could improve the disease resistance of *Litopenaeus vannamei* by regulating the oxidative phosphorylation, Aquac. Reports 41 (2025) 102687. https://doi.org/10.1016/j.aqrep.2025.102687.

[58] M. Tlusty, A. Rhyne, J.T. Szczebak, B. Bourque, J.L. Bowen, G. Burr, C.J. Marx, L. Feinberg, A transdisciplinary approach to the initial validation of a single cell protein as an alternative protein source for use in aquafeeds, PeerJ 2017 (2017) 1–19. https://doi.org/10.7717/peerj.3170.

[59] D. Wu, Z. Fan, X. Zheng, J. Li, M. Zhou, H. Zhang, H. Xu, S. Xie, L. Wang, Evaluation of four novel non-grain protein sources completely replacing soybean meal on growth performance, serum biochemistry, amino acid transport and intestinal health of grass carp (*Ctenopharyngodon idella*) at different water temperatures, Fish Shellfish Immunol. 153 (2024) 109807. https://doi.org/10.1016/j.fsi.2024.109807.

[60] R. Xue, H. Li, S. Liu, Z. Hu, Q. Wu, H. Ji, Substitution of soybean meal with *Clostridium autoethanogenum* protein in grass carp (*Ctenopharygodon idella*) diets: Effects on growth performance, feed utilization, muscle nutritional value and sensory characteristics, Anim. Feed Sci. Technol. 295 (2023) 115547. https://doi.org/10.1016/j.anifeedsci.2022.115547.

[61] H. Yu, H. Liang, M. Longshaw, J. Wang, X. Ge, M. Ren, L. Zhang, Methanotroph (*Methylococcus capsulatus*, Bath) bacteria meal (FeedKind®) could effectively improve the growth, apparent digestibility coefficient, blood biochemical parameters, antioxidant indices of juvenile Jian carp (*Cyprinus carpio* var. Jian), Anim. Feed Sci. Technol. 288 (2022) 115293. https://doi.org/10.1016/j.anifeedsci.2022.115293.

[62] H. Yuan, N. Hu, Y. Zheng, C. Hou, B. Tan, L. Shi, S. Zhang, A Comparison of Three Protein Sources Used in Medium-Sized *Litopenaeus vannamei*: Effects on Growth, Immunity, Intestinal Digestive Enzyme Activity, and Microbiota Structure, Fishes 8 (2023) 449. https://doi.org/10.3390/fishes8090449.

[63] Y. Chen, S. Chi, S. Zhang, X. Dong, Q. Yang, H. Liu, B. Tan, S. Xie, Evaluation of Methanotroph (*Methylococcus capsulatus*, Bath) bacteria meal on body composition, lipid metabolism, protein synthesis and muscle metabolites of Pacific white shrimp (*Litopenaeus vannamei*), Aquaculture 547 (2022) 737517. https://doi.org/10.1016/j.aquaculture.2021.737517.

[64] K. Chen, G. Sagada, B. Xu, Y. Liu, L. Zheng, A.F. Tegomo, Y. Yang, Y. Sun, J. Wang, M. Longshaw, Q. Shao, Evaluation of methanotroph (*Methylococcus capsulatus*, Bath) bacteria meal as an alternative protein source for growth performance, digestive enzymes, and health status of Pacific, Aquac. Int. 30 (2022) 1693–1710. https://doi.org/10.1007/s10499-022-00869-9.

[65] J. Chen, H. Wang, H. Yuan, N. Hu, F. Zou, C. Li, L. Shi, B. Tan, S. Zhang, Effects of dietary *Clostridium autoethanogenum* protein on the growth, disease resistance, intestinal digestion, immunity and microbiota structure of *Litopenaeus vannamei* reared at different water salinities, Front. Immunol. 13 (2022) 1–18. https://doi.org/10.3389/fimmu.2022.1034994.

[66] Y. Chen, C. Zheng, X. Zhang, X. Li, X. Yao, G. He, J. Cao, S. Chi, S. Zhang, B. Tan, S. Xie, Evaluation of ratios of fish-to-soybean oils on growth, lipid and cholesterol metabolism and muscle metabolites of Pacific white shrimp (*Litopenaeus vannamei*) fed low fishmeal diets containing *Clostridium autoethanogenum* protein, Aquac. Reports 27 (2022) 101417. https://doi.org/10.1016/j.aqrep.2022.101417.

[67] J. Chen, H. Wang, H. Yuan, N. Hu, Y. Zheng, Y. Hu, S. Zhang, Transcriptomic analysis reveals the immunodulatory role of *Clostridium autoethanogenum* protein on *Litopenaeus vannamei* under variable salinity conditions, Anim. Nutr. J. (2025). https://doi.org/10.1016/j.aninu.2025.04.010.

[68] N. Bai, Q. Li, S. Pan, Z. Qi, W. Deng, M. Gu, Effects of *Clostridium autoethanogenum* protein on growth performance, intestine and liver health of turbot *Scophthalmus maximus* during grow-out phase, Aquac. Reports 36 (2024) 102076. https://doi.org/10.1016/j.aqrep.2024.102076.

[69] G. Marit Berge, G. Baeverfjord, A. Skrede, T. Storebakken, Bacterial protein grown on natural gas as protein source in diets for Atlantic salmon, *Salmo salar*, in saltwater, Aquaculture 244 (2005) 233–240. https://doi.org/10.1016/j.aquaculture.2004.11.017.

[70] M.A. Rhodes, Y. Zhou, D.A. Davis, Use of Dried Fermented Biomass as a Fish Meal Replacement in Practical Diets of Florida Pompano, *Trachinotus carolinus*, J. Appl. Aquac. 27 (2015) 29–39. https://doi.org/10.1080/10454438.2014.959834.

[71] P. Yang, X. Li, W. Yao, M. Li, Y. Wang, X. Leng, Dietary Effect of *Clostridium autoethanogenum* Protein on Growth, Intestinal Histology and Flesh Lipid Metabolism of Largemouth Bass (*Micropterus salmoides*) Based on Metabolomics, Metabolites 12 (2022) 1088. https://doi.org/10.3390/metabo12111088.

[72] P. Yang, X. Li, B. Song, M. He, C. Wu, X. Leng, The potential of *Clostridium autoethanogenum*, a new single cell protein, in substituting fish meal in the diet of largemouth bass (*Micropterus salmoides*): Growth, feed utilization and intestinal histology, Aquac. Fish. 8 (2023) 67–75. https://doi.org/10.1016/j.aaf.2021.03.003.

[73] Y. Wu, S. Tian, J. Yuan, Z. Zhang, H. Zhou, W. Gao, W. Zhang, K. Mai, Effects of *Clostridium autoethanogenum* protein as substitute for dietary fishmeal on the growth, feed utilization, intestinal health and muscle quality of large yellow croaker *Larimichthys crocea*, Aquaculture 561 (2022) 738591. https://doi.org/10.1016/j.aquaculture.2022.738591.

[74] M. Cai, L. Yang, L. Zhong, S. Xie, Y. Hu, Response patterns and community assembly processes of gut microbiota in grass carp subjected to various protein sources and their implications for growth and metabolism, Fish Physiol. Biochem. 51 (2025) 1–83. https://doi.org/10.1007/s10695-025-01498-8.

[75] L. Yang, M. Cai, S. Xie, Y. Hu, J. Zhang, Evaluation of soybean meal replacement by *Clostridium autoethanogenum* protein in diets for juvenile grass carp (*Ctenopharyngodon idellus*), Aquaculture 592 (2024) 741218. https://doi.org/10.1016/j.aquaculture.2024.741218.

[76] W. Yao, P. Yang, X. Zhang, X. Xu, C. Zhang, X. Li, X. Leng, Effects of replacing dietary fish meal with *Clostridium autoethanogenum* protein on growth and flesh quality of Pacific white shrimp (*Litopenaeus vannamei*), Aquaculture 549 (2022) 737770. https://doi.org/10.1016/j.aquaculture.2021.737770.

[77] Y. Huangfu, P. Qu, D. Liu, X. Wang, D. Huang, Z. Wu, J. Sahandi, K. Mai, W. Zhang, Protein requirements of large yellow croaker *Larimichthys crocea* depends on protein sources from the perspective of growth performance, digestive and absorptive enzyme activities, intestinal and liver histology, Aquac. Reports 36 (2024) 102139. https://doi.org/10.1016/j.aqrep.2024.102139.

[78] X. Liu, L. Li, Y. Huang, C. Wang, Interactions between different single protein and feeding level on growth performance, hepatic lipid metabolism and health of largemouth bass (*Micropterus salmoides*), Anim. Feed Sci. Technol. 325 (2025) 116342. https://doi.org/10.1016/j.anifeedsci.2025.116342.

[79] Z. Qi, N. Bai, Q. Li, S. Pan, M. Gu, Dietary fishmeal replacement by *Clostridium autoethanogenum* protein meal influences the nutritional and sensory quality of turbot (*Scophthalmus maximus*) via the TOR/AAR/AMPK pathways, Anim. Nutr. 18 (2024) 84–95. https://doi.org/10.1016/j.aninu.2024.04.012.

[80] Y. Wang, L. Li, Y. Huang, C. Wang, Triggering compensatory growth by completely replacing fishmeal with novel protein sources in the diets of juvenile largemouth bass (*Micropterus salmoides*): Effects on growth performance and liver health, Aquac. Fish. (2025) 1–11. https://doi.org/10.1016/j.aaf.2025.03.001.

[81] Z. Wu, X. Yu, J. Guo, Y. Fu, Y. Guo, M. Pan, W. Zhang, K. Mai, Replacement of dietary fish meal with *Clostridium autoethanogenum* protein on growth performance, digestion, mTOR pathways and muscle quality of abalone *Haliotis discus* hannai, Aquaculture 553 (2022) 738070. https://doi.org/10.1016/j.aquaculture.2022.738070.

[82] J. Zhang, Y. Dong, K. Song, L. Wang, X. Li, K. Lu, B. Tan, C. Zhang, Substituting Fish Meal with a Bacteria Protein (*Clostridium autoethanogenum* Protein) Derived from Industrial-Scale Gas Fermentation: Effects on Growth and Gut Health of Juvenile Large Yellow Croakers (*Larimichthys crocea*), Fishes 7 (2022) 228. https://doi.org/10.3390/fishes7050228.
