# Supplementary figures and images for: Bacterial Single‐Cell Proteins as Sustainable Aquafeeds: A Meta‐Analysis of Growth, Physiological Homeostasis, and Antioxidant Capacity
Source: Aquac Nutr. 2026 Apr 25;2026:4548847. doi: 10.1155/anu/4548847 (PMC13110275; doi:10.1155/anu/4548847)

## Slide 1
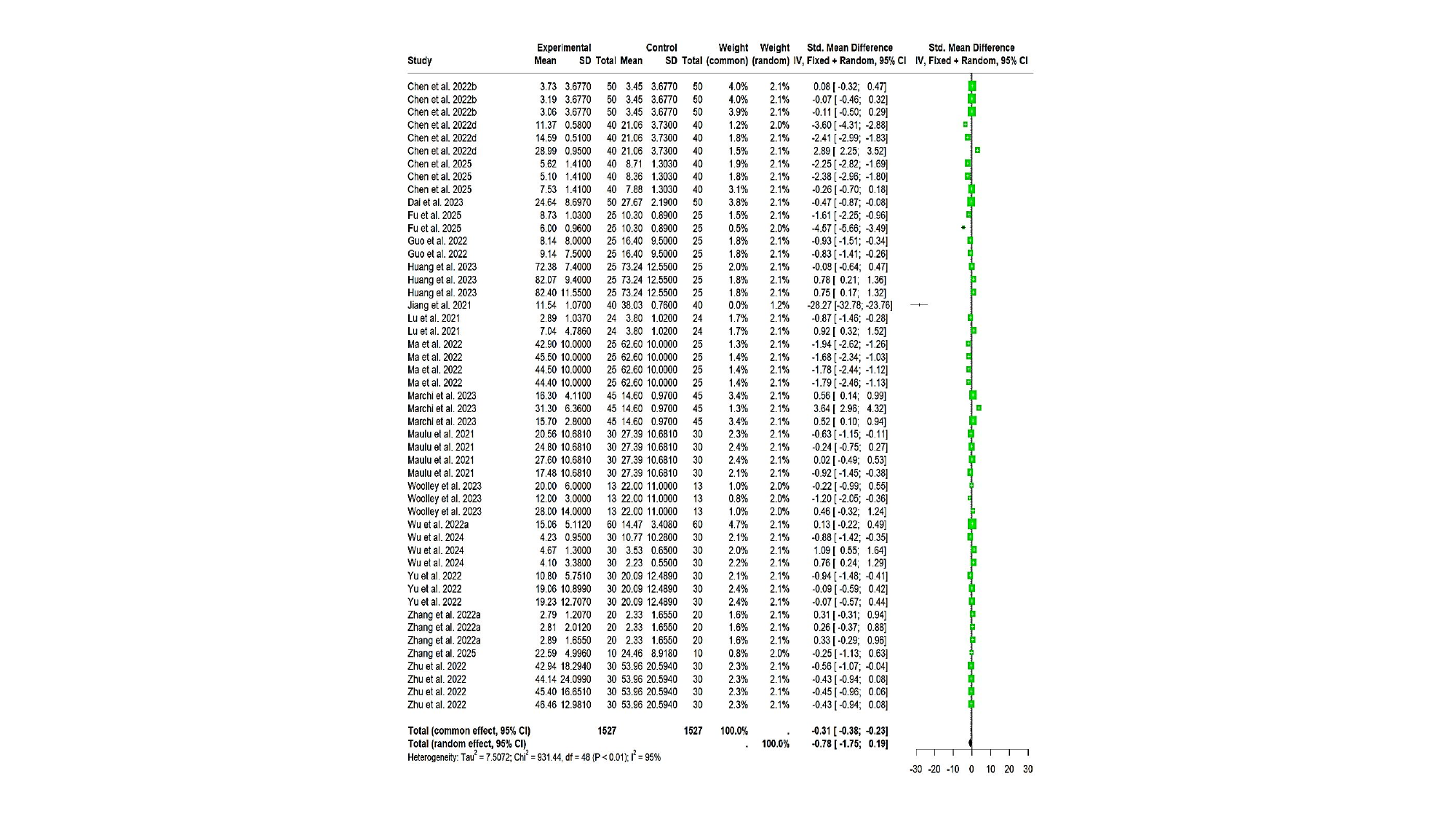

Supplement: Supplementary file 1 — Supporting Information Table S1: Detailed parameters of 74 independent studies for the specific growth (SGR) of carnivorous species. This table includes the author names and year of publication, sample size, mean, and standard deviation of experimental and control groups, respectively. Table S2: Detailed parameters of 84 independent studies for the SGR of omnivorous and herbivorous species. This table includes the author names and year of publication, sample size, mean, and standard deviation of experimental and control groups, respectively. Table S3: Detailed parameters of 75 independent studies for the feed conversion ratio (FCR) of carnivorous species. This table includes the author names and year of publication, sample size, mean, and standard deviation of experimental and control groups, respectively. Table S4: Detailed parameters of 89 independent studies for the FCR of omnivorous and herbivorous species. This table includes the author names and year of publication, sample size, mean, and standard deviation of experimental and control groups, respectively. Table S5: Detailed parameters of 65 independent studies for the SR of carnivorous species. This table includes the author names and year of publication, sample size, mean, and standard deviation of experimental and control groups, respectively. Table S6: Detailed parameters of 77 independent studies for the SR of omnivorous and herbivorous species. This table includes the author names and year of publication, sample size, mean, and standard deviation of experimental and control groups, respectively. Table S7: Detailed parameters of 88 independent studies for the hepatosomatic index (HSI) of aquaculture species. This table includes the author names and year of publication, sample size, mean, and standard deviation of experimental and control groups, respectively. Table S8: Detailed parameters of 67 independent studies for the viscerosomatic index (VSI) of aquaculture species. This table includes the author na [file ANU-2026-4548847-s001.zip › Supplementary Figure 1.pptx]

## Slide 1
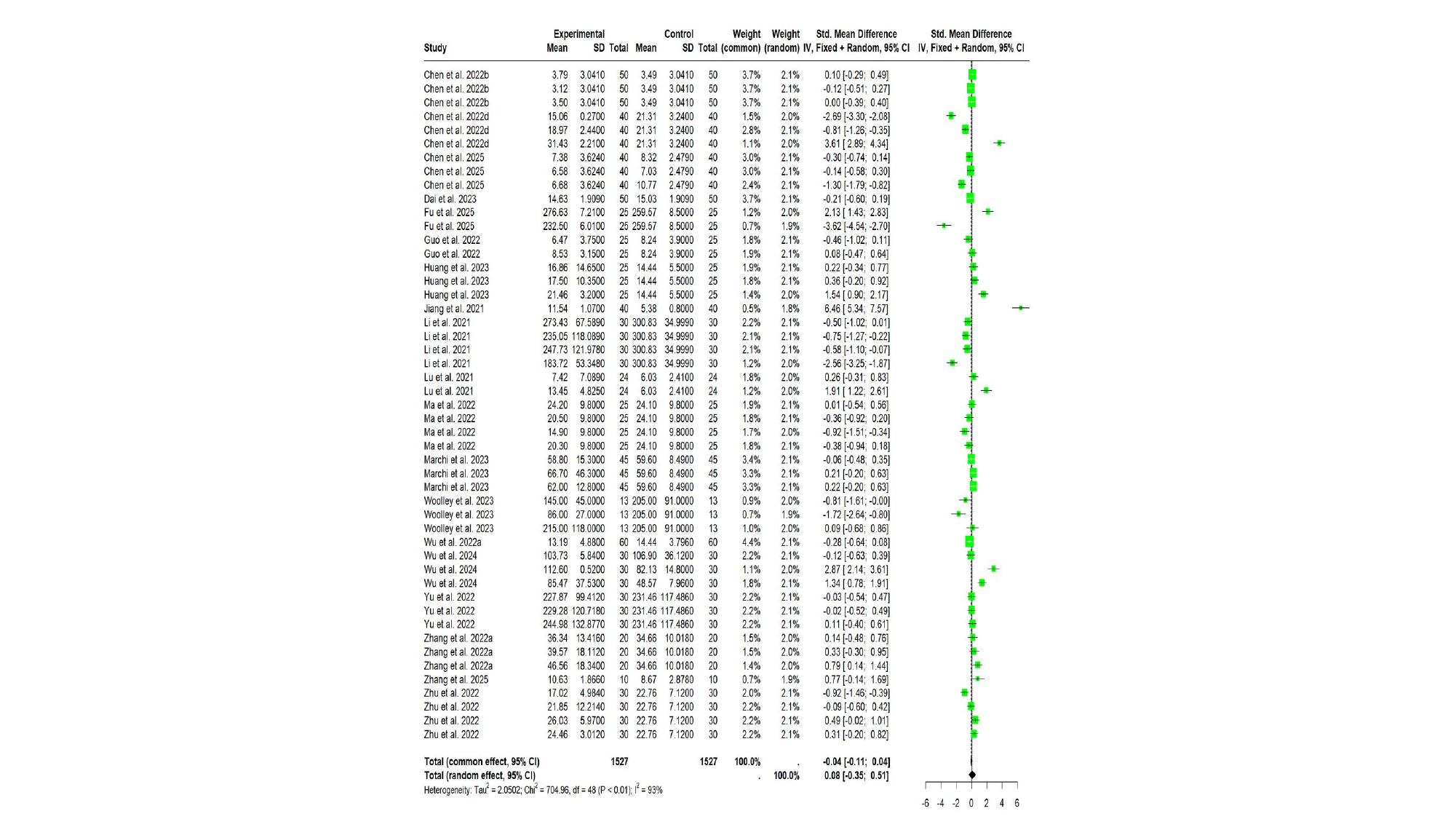

Supplement: Supplementary file 1 — Supporting Information Table S1: Detailed parameters of 74 independent studies for the specific growth (SGR) of carnivorous species. This table includes the author names and year of publication, sample size, mean, and standard deviation of experimental and control groups, respectively. Table S2: Detailed parameters of 84 independent studies for the SGR of omnivorous and herbivorous species. This table includes the author names and year of publication, sample size, mean, and standard deviation of experimental and control groups, respectively. Table S3: Detailed parameters of 75 independent studies for the feed conversion ratio (FCR) of carnivorous species. This table includes the author names and year of publication, sample size, mean, and standard deviation of experimental and control groups, respectively. Table S4: Detailed parameters of 89 independent studies for the FCR of omnivorous and herbivorous species. This table includes the author names and year of publication, sample size, mean, and standard deviation of experimental and control groups, respectively. Table S5: Detailed parameters of 65 independent studies for the SR of carnivorous species. This table includes the author names and year of publication, sample size, mean, and standard deviation of experimental and control groups, respectively. Table S6: Detailed parameters of 77 independent studies for the SR of omnivorous and herbivorous species. This table includes the author names and year of publication, sample size, mean, and standard deviation of experimental and control groups, respectively. Table S7: Detailed parameters of 88 independent studies for the hepatosomatic index (HSI) of aquaculture species. This table includes the author names and year of publication, sample size, mean, and standard deviation of experimental and control groups, respectively. Table S8: Detailed parameters of 67 independent studies for the viscerosomatic index (VSI) of aquaculture species. This table includes the author na [file ANU-2026-4548847-s001.zip › Supplementary Figure 2.pptx]

## Slide 1
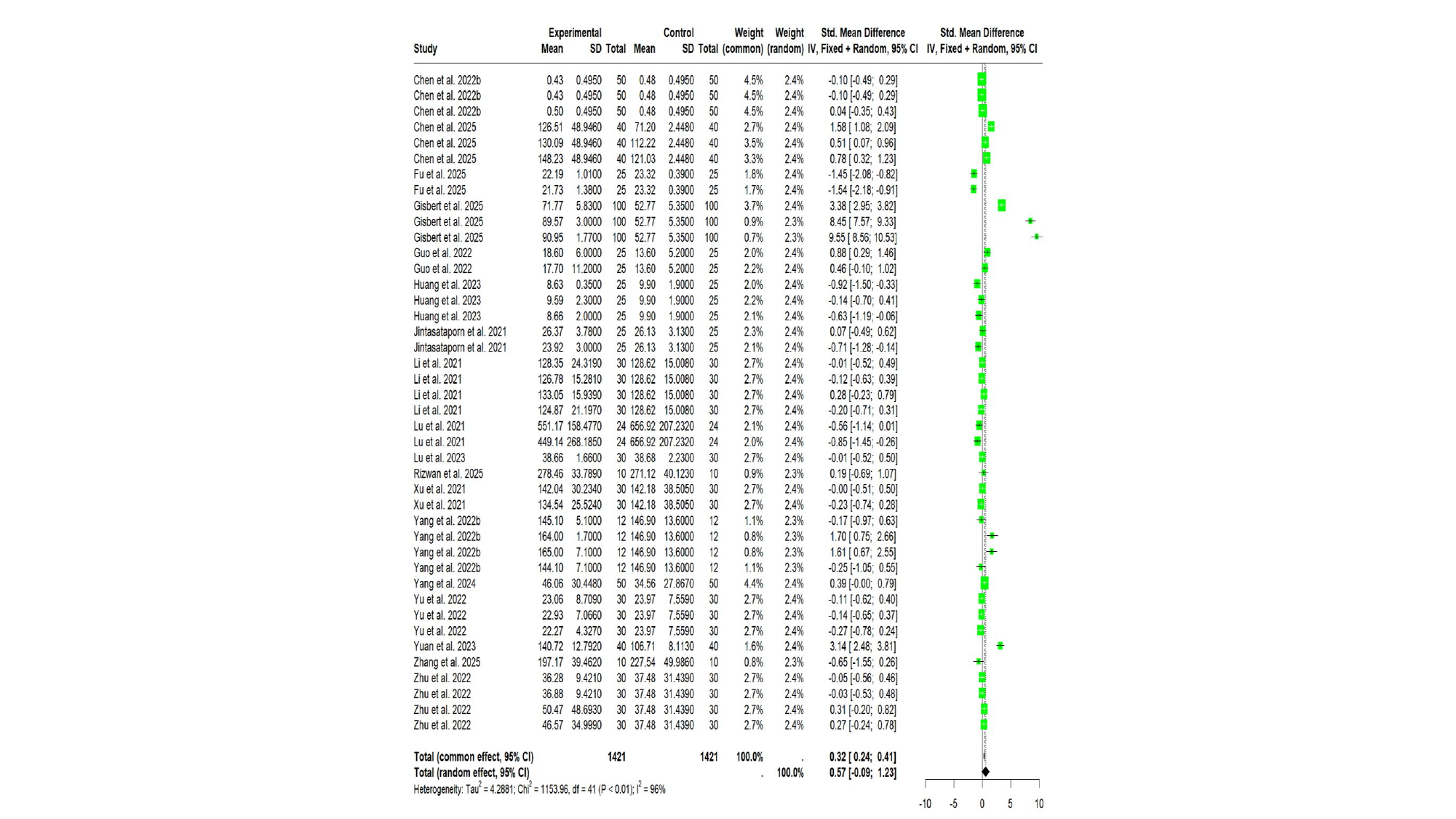

Supplement: Supplementary file 1 — Supporting Information Table S1: Detailed parameters of 74 independent studies for the specific growth (SGR) of carnivorous species. This table includes the author names and year of publication, sample size, mean, and standard deviation of experimental and control groups, respectively. Table S2: Detailed parameters of 84 independent studies for the SGR of omnivorous and herbivorous species. This table includes the author names and year of publication, sample size, mean, and standard deviation of experimental and control groups, respectively. Table S3: Detailed parameters of 75 independent studies for the feed conversion ratio (FCR) of carnivorous species. This table includes the author names and year of publication, sample size, mean, and standard deviation of experimental and control groups, respectively. Table S4: Detailed parameters of 89 independent studies for the FCR of omnivorous and herbivorous species. This table includes the author names and year of publication, sample size, mean, and standard deviation of experimental and control groups, respectively. Table S5: Detailed parameters of 65 independent studies for the SR of carnivorous species. This table includes the author names and year of publication, sample size, mean, and standard deviation of experimental and control groups, respectively. Table S6: Detailed parameters of 77 independent studies for the SR of omnivorous and herbivorous species. This table includes the author names and year of publication, sample size, mean, and standard deviation of experimental and control groups, respectively. Table S7: Detailed parameters of 88 independent studies for the hepatosomatic index (HSI) of aquaculture species. This table includes the author names and year of publication, sample size, mean, and standard deviation of experimental and control groups, respectively. Table S8: Detailed parameters of 67 independent studies for the viscerosomatic index (VSI) of aquaculture species. This table includes the author na [file ANU-2026-4548847-s001.zip › Supplementary Figure 3.pptx]

## Slide 1
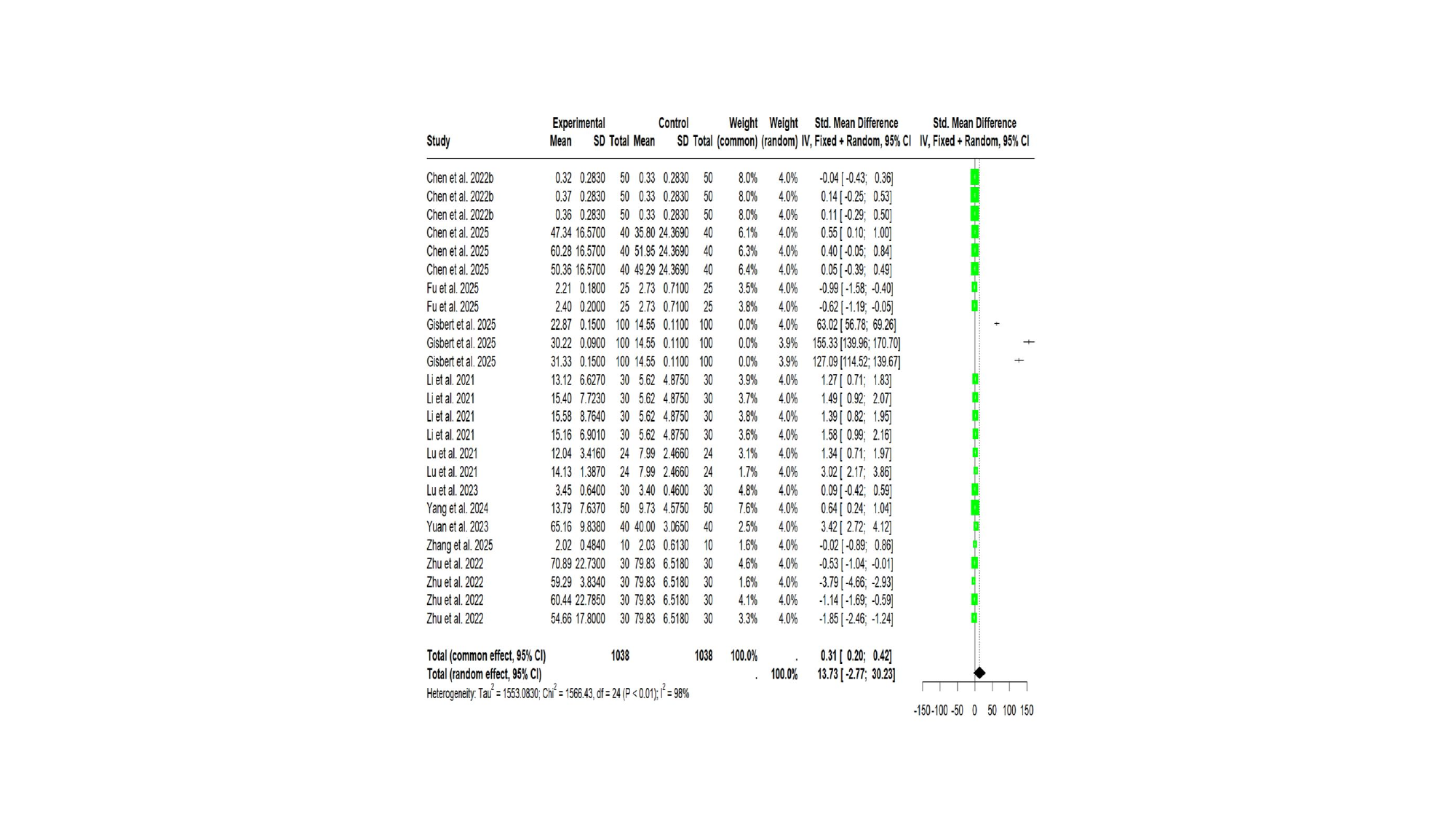

Supplement: Supplementary file 1 — Supporting Information Table S1: Detailed parameters of 74 independent studies for the specific growth (SGR) of carnivorous species. This table includes the author names and year of publication, sample size, mean, and standard deviation of experimental and control groups, respectively. Table S2: Detailed parameters of 84 independent studies for the SGR of omnivorous and herbivorous species. This table includes the author names and year of publication, sample size, mean, and standard deviation of experimental and control groups, respectively. Table S3: Detailed parameters of 75 independent studies for the feed conversion ratio (FCR) of carnivorous species. This table includes the author names and year of publication, sample size, mean, and standard deviation of experimental and control groups, respectively. Table S4: Detailed parameters of 89 independent studies for the FCR of omnivorous and herbivorous species. This table includes the author names and year of publication, sample size, mean, and standard deviation of experimental and control groups, respectively. Table S5: Detailed parameters of 65 independent studies for the SR of carnivorous species. This table includes the author names and year of publication, sample size, mean, and standard deviation of experimental and control groups, respectively. Table S6: Detailed parameters of 77 independent studies for the SR of omnivorous and herbivorous species. This table includes the author names and year of publication, sample size, mean, and standard deviation of experimental and control groups, respectively. Table S7: Detailed parameters of 88 independent studies for the hepatosomatic index (HSI) of aquaculture species. This table includes the author names and year of publication, sample size, mean, and standard deviation of experimental and control groups, respectively. Table S8: Detailed parameters of 67 independent studies for the viscerosomatic index (VSI) of aquaculture species. This table includes the author na [file ANU-2026-4548847-s001.zip › Supplementary Figure 4.pptx]
